# DP1 Receptor Blockade Attenuates Microglial Senescence and Cognitive Decline Caused by PTGDS in Exosomes From Aged Brains

**DOI:** 10.1111/acel.70228

**Published:** 2025-09-19

**Authors:** Yaru Liu, Pan Liao, Bo Yan, Dai Li, Shishuang Zhang, Wei Zhang, Zexi Jia, Zihan Zhang, Han Gao, Qiang Liu, Fanglian Chen, Ping Lei, Zhenyu Yin

**Affiliations:** ^1^ Department of Geriatrics Tianjin Medical University General Hospital Tianjin China; ^2^ Tianjin Institute of Geriatrics Tianjin China; ^3^ School of Medicine Nankai University Tianjin China; ^4^ Department of Neurology, National Clinical Research Center for Geriatric Diseases, Xuanwu Hospital Capital Medical University Beijing China

**Keywords:** aging, DP1 receptors, exosomes, microglia, neuroinflammation

## Abstract

Aging leads to neurodegenerative diseases, such as cognitive decline, which are induced by persistent chronic low‐grade inflammation in the brain driven by microglial activation. However, whether and how brain‐derived exosomes from aged mice (A‐exo) induce a pro‐inflammatory state and cellular senescence in microglia within the aging brain is poorly understood. Here, we report that brain‐derived exosomes from aged mice (A‐exo) cause cognitive decline in normal young mice, inducing microglial overactivation, lipid droplet accumulation, and senescence‐associated secretory phenotype (SASP) secretion. This abnormal microglial activity arises from the elevated expression of PTGDS in A‐exo due to mouse aging, resulting in increased central and peripheral D‐prostanoid receptor 1 (DP1) ligand PGD2 levels, which subsequently leads to sustained DP1 signaling activation. Consequently, this process promotes myeloid cell infiltration, cellular senescence, and cognitive decline by generating a senescent, pro‐inflammatory microglial phenotype. Blocking the DP1 receptor ameliorates A‐exo‐mediated microglial overactivation, myeloid cell infiltration, and cellular senescence. Strikingly, DP1 receptor blockade improves cellular senescence, neuroinflammation, and cognitive decline in aged mice. Our findings reveal a systemic mechanism underlying the sustained activation of microglia following brain aging, paving the way for improving chronic neuroinflammation, cellular senescence, and cognitive decline associated with aging.

## Graphical abstract

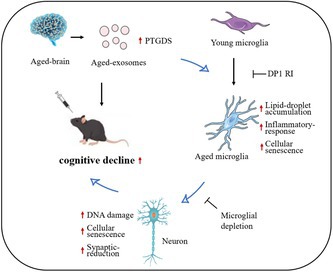

## 1 Introduction

Aging is a major risk factor for cognitive decline and neurodegenerative diseases, characterized by chronic neuroinflammation and synaptic dysfunction (López‐Otín et al. 2023). Recent reviews highlight that age‐related cognitive impairment involves complex interactions between cellular senescence, oxidative stress, and immune dysregulation (A. G. Thompson et al. 2016). Notably, chronic low‐grade inflammation in the aging brain, termed “inflammaging,” drives microglial activation and neuronal damage, contributing to pathologies such as Alzheimer's disease (AD) and Parkinson's disease (PD) (Deczkowska et al. 2018). While genetic and environmental factors are well studied, emerging evidence underscores the role of systemic mediators, including exosomes, in propagating age‐related neurodegeneration (H. Zhang, Lin, et al. 2021).

Exosomes, nanosized extracellular vesicles, have emerged as critical mediators of intercellular communication in neurodegenerative disorders. Studies reveal that exosomes from aged brains or serum carry pro‐inflammatory proteins, miRNAs, and lipids that exacerbate neuroinflammation and synaptic loss (Goetzl et al. 2016; Ruan et al. 2021). For instance, exosomes derived from the plasma of AD patients propagate tau pathology and impair neuronal plasticity (A. G. Thompson et al. 2016). Similarly, aged serum exosomes worsen stroke outcomes by priming microglial phagocytosis through complement signaling (H. Zhang, Lin, et al. 2021). These findings highlight the dual role of exosomes as both pathogenic vectors and potential therapeutic targets in age‐related cognitive decline.

Microglia, the brain's resident immune cells, are central to maintaining neural homeostasis but become dysregulated with aging. Activated microglia adopt a pro‐inflammatory phenotype, releasing cytokines and promoting synaptic pruning, which disrupts cognitive function (Hammond et al. 2019; E. E. Spangenberg et al. 2016). Single‐cell RNA sequencing studies demonstrate that aged microglia exhibit senescence‐associated secretory phenotypes (SASP), marked by lipid droplet accumulation and impaired phagocytosis (Deczkowska et al. 2018). Furthermore, microglial depletion or modulation of inflammatory pathways (e.g., TNF receptor signaling) ameliorates cognitive deficits in aging models (E. E. Spangenberg et al. 2016; T. Zhang, Yu, et al. 2021). These insights underscore microglia as pivotal drivers of age‐related neuroinflammation and cognitive decline. Currently, there is no effective approach to ameliorate microglial hyperactivation and proinflammatory mediator secretion during brain aging. The impact of exosomes secreted by the aging brain on central immune cells remains unclear. As the primary immune cells in the brain, microglia exhibit poorly understood specific responses to exosomes secreted by the aging brain, and the potential effects of these exosomes on age‐related neuroinflammation and cognitive decline are largely unknown.

Prostaglandin D2 (PGD2), a lipid mediator synthesized by PTGDS, signals through the DP1 receptor to regulate inflammation and cellular senescence. Recent studies implicate PGD2‐DP1 signaling in neuroinflammatory diseases, where elevated PGD2 levels correlate with microglial activation and neuronal damage (I. Mohri et al. 2006). In aging brains, PTGDS overexpression in astrocytes and oligodendrocytes amplifies PGD2 production, exacerbating neuroinflammation (Kihara et al. 2015). Notably, DP1 receptor inhibition reduces microglial SASP secretion and improves cognitive function in AD models (Wallace et al. 2022). These findings position the PGD2‐DP1 axis as a promising therapeutic target for age‐related neurodegeneration.

In this study, we found that the sustained activation of microglial DP1 signaling was induced by brain‐derived exosomes from aged mice. The progressive accumulation of PGD_2_ ligands in both the circulation and the central nervous system led to promoting a proinflammatory microglial phenotype, thereby driving the progression of neuroinflammation during aging. This process could be therapeutically targeted through the administration of DP1 receptor inhibitors. Our findings provide a theoretical basis for alleviating aging‐related neuroinflammation and cognitive decline. DP1 receptor inhibitors hold promise as preventive measures against age‐related neurofunctional impairments and cellular senescence, positioning them as promising therapeutic targets for age‐related neurological disorders.

## 2 Results

### 2.1 Brain‐Derived Exosomes From Aged Mice (A‐Exo) Lead to Cognitive Decline in Normal Young Mice

**FIGURE 1:**
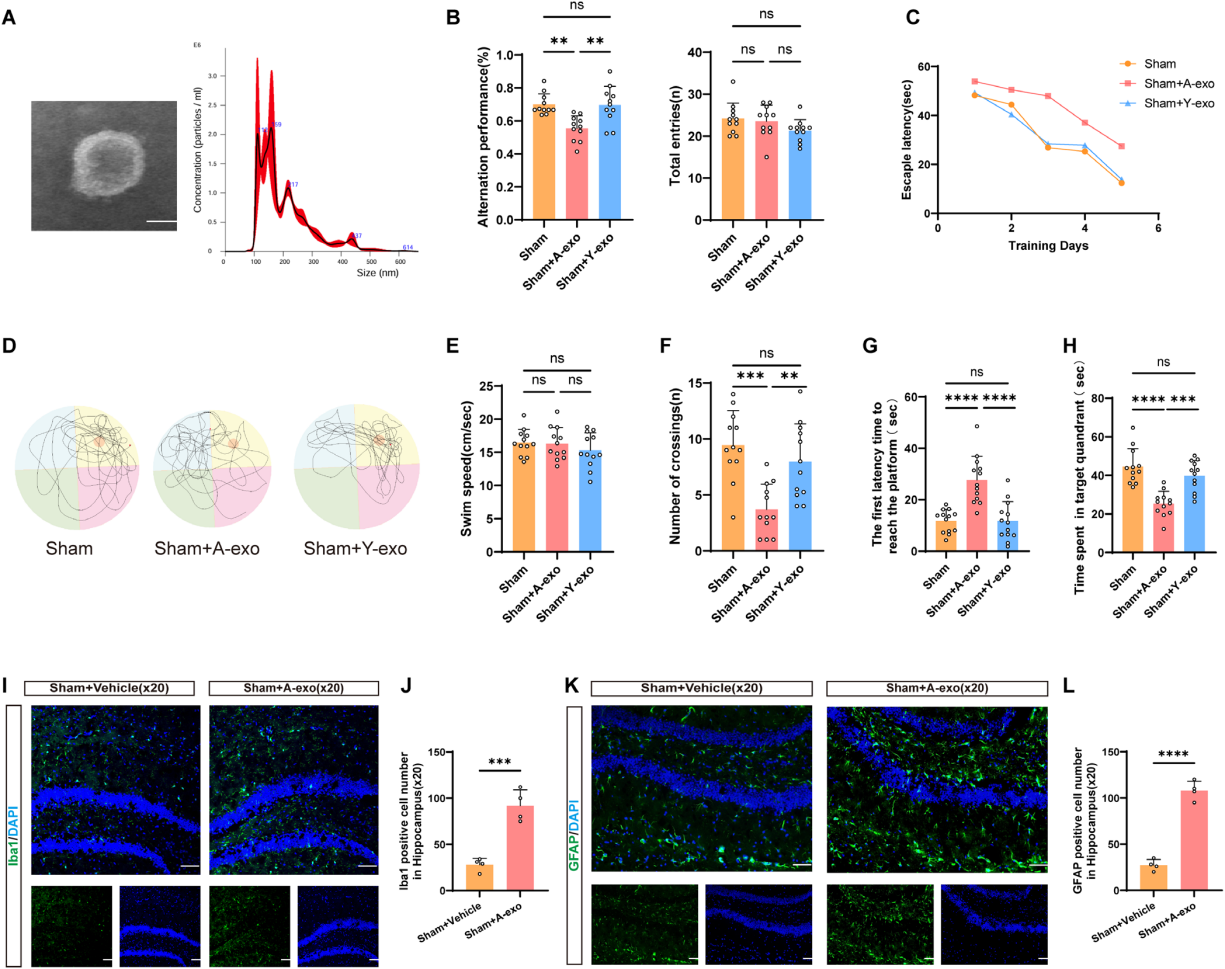
Brain‐derived exosomes from aged mice (A‐exo) lead to cognitive decline in normal young mice. (A) TEM image of purified exosomes from brain‐derived exosomes from aged mice, characterization of purified exosomes using NTA. Scale bars, 100 nm. (B) Y maze is performed on day 36 after exosomes injection into the hippocampus (*n* = 11). (C) Escape latency in the training phase (*n* = 12). (D) Representative traces in the Morris water maze. (E) Swim speed during the probe trial of MWM. (F) Number of crossings during the probe trial of MWM. (G) The first latency time to reach the platform during the probe trial of MWM. (H) Time spent in the target quadrant during the probe trial of MWM. (I) Representative immunofluorescence staining of Iba1‐positive cells. Green indicates Iba1‐positive staining, and blue indicates positive DAPI nuclear staining. Scale bar = 50 μm. (J) Quantification of the number of Iba1‐positive cell numbers (*n* = 4 mice per group). (K) Representative immunofluorescence staining of GFAP‐positive cells. Green indicates GFAP‐positive staining, and blue indicates positive DAPI nuclear staining. Scale bar = 50 μm. (L) Quantification of the number of GFAP‐positive cell numbers (*n* = 4 mice per group). (Data were represented as the mean ± SD. **p < 0.01, ***p < 0.001, and ****p < 0.0001. A‐exo, Brain‐derived exosomes from aged mice; Y‐exo, Brain‐derived exosomes from young mice.)

To investigate the impact of brain‐derived exosomes from aged mice on the cognitive function of normal young mice, we collected exosomes sourced from the brains of aged mice and employed transmission electron microscopy (TEM) and Nanoparticle Tracking Analysis (NTA) to measure their morphology and size (Figure 1A). Subsequently, these brain‐derived exosomes from aged mice were injected into the hippocampus of normal young mice, and we assessed the Y‐maze performance of mice at 36 days and evaluated the effect of A‐exo on recognition memory by calculating alternation performance. Compared to the Sham group, the alternation performance of the Sham+A‐exo group significantly decreased, indicating impaired memory. In comparison to the Sham group, there was no decline in cognitive function in the Sham+Y‐exo group (Figure 1B).

Between days 37 and 42 after injection of exosomes in the hippocampus, the water maze was performed. Mice in the Sham+A‐exo group exhibited impaired spatial learning and memory compared to the Sham group. In comparison to the Sham group, there was no decline in cognitive function in the Sham+Y‐exo group, as evidenced by their swimming paths and learning curves (Figure 1C,D). During the training phase, mice of the Sham+Y‐exo group showed a decrease in escape latency, while mice in the Sham+A‐exo group exhibited significantly longer escape latencies compared to sham‐treated mice (Figure 1C). In the probe test, the number of crossings over the target quadrant platform significantly decreased in the Sham+A‐exo group (Figure 1F), while the latency to first platform arrival increased (Figure 1G), indicating impaired spatial learning and memory function. Compared to the Sham+A‐exo group, mice in the Sham+Y‐exo group spent significantly more time in the target quadrant (Figure 1H) and had reduced latency to first platform arrival (Figure 1G). Additionally, there were no significant differences in average swimming speed among the three groups of mice (Figure 1E). Immunofluorescence analysis demonstrated significant upregulation of Iba1 and GFAP expression in the hippocampal regions of Sham+A‐exo group brains compared with Sham+Vehicle controls (Figure 1I–L). Additionally, Western blot (WB) results showed that compared with the control group, A‐exo reduced the expression of the synaptic‐associated protein PSD95 in the brain (Figure S1A,B). In conclusion, mice treated with A‐exo showed impaired spatial learning and memory function compared to the Sham group. Conversely, mice treated with Y‐exo demonstrated no decline in spatial learning and memory function.

### 2.2 PTGDS is Highly Expressed in A‐Exo, and the DP1 Ligands Continue to Increase Following Brain Aged

**FIGURE 2:**
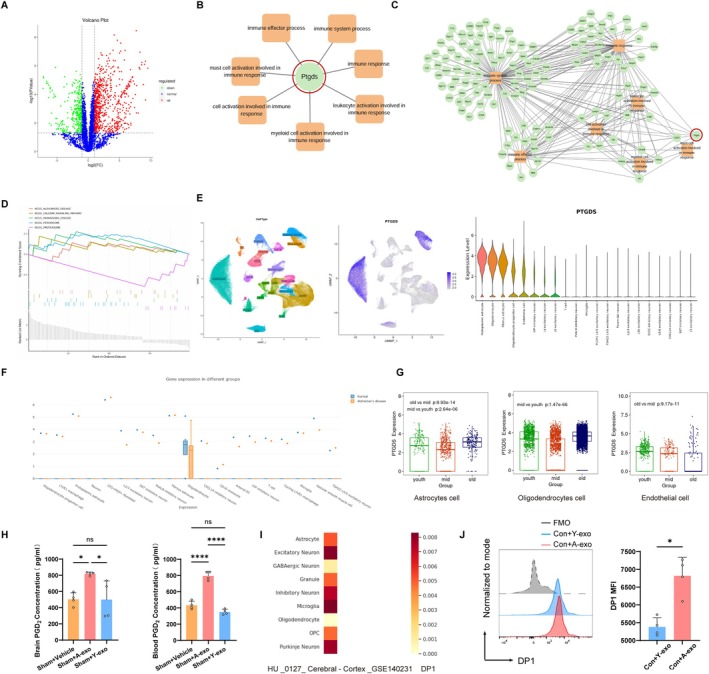
PTGDS is highly expressed in A‐exo, and the DP1 ligands continue to increase following brain aged. (A) Volcano plot of DEGs between A‐exo and Y‐exo groups. (B) PPI network of DEGs of the signaling pathways associated with PTGDS. (C) Pathway enrichment analysis was performed for differentially expressed genes (DEGs) associated with PTGDS. (D) The significant GSEA sets in KEGG pathways. (E) UMAP plot of PTGDS‐expressing cell types in the brain of the DISCO database (left). Violin diagram of various cell types expressing PTGDS (right). (F) The content of PTGDS expressed in different cells of Alzheimer's disease in the DISCO database. (G) Expression of PTGDS in youth, mid group, and old groups in the ScAgeAnno database. (H) ELISA of PGD2 content in mouse brain tissue and blood. *n* = 4 mice per group. (I) Expression of DP1 in HU_0127_Cerebral‐Cortex_GSE140231 in the HUSCH database. (J) Flow cytometry histogram of microglia DP1 expression after 24 h of exosomes treatment. Quantification of DP1. *n* = 4 per group. Data were representative of four independent experiments. (Data were represented as the mean ± SD. *p < 0.05, ****p < 0.0001.)

To investigate the causes of cognitive decline in normal young mice induced by brain‐derived exosomes from aged mice, we conducted proteomic sequencing of aged mouse brain‐derived exosomes (A‐exo) and young mouse brain‐derived exosomes (Y‐exo). Proteomic sequencing results revealed that, based on the selection criteria of |log2(FC)| ≥ 1.00 and *p* value ≤ 0.05 during the differential expression protein analysis, there were 1066 differentially expressed proteins between A‐exo and Y‐exo. Compared with Y‐exo, A‐exo had 726 upregulated proteins and 340 downregulated proteins (Figure 2A). To further elucidate the function of the significantly differentially expressed protein PTGDS, bioinformatics enrichment analysis was performed, revealing that PTGDS is primarily involved in immune system processes and immune responses (Figure 2B). Figure 2C displays the differentially expressed proteins related to immune system processes and immune responses. GSEA enrichment analysis of differential proteins found that the top five KEGG enrichment analyses were ALZHEIMERS_DISEASE, CALCIUM_SIGNALING_PATHWAY, PARKINSONS_DISEASE, PEROXISOME, and PROTEASOME (Figure 2D), which are significantly related to cognitive function. The Deeply Integrated human Single‐Cell Omics data (DISCO) database indicated that the main cells expressing PTGDS in the brain include astrocytes, oligodendrocytes, endothelial cells, and neurons (Figure 2E). In patients with Alzheimer's disease, PTGDS is primarily expressed in oligodendrocytes (Figure 2F). The AgeAnno single‐cell database showed that in the elderly, PTGDS is mainly expressed in astrocytes, oligodendrocytes, and endothelial cells (Figure 2G). To ascertain whether A‐exo leads to an increase in PGD2, the ligand for DP1, in the brain and periphery of mice, we conducted Elisa assays for PGD2 in mouse brain tissue and peripheral blood after exosome intervention. The results indicated that the A‐exo intervention group significantly increased PGD2 levels in both mouse brain tissue and peripheral blood (Figure 2H).

Data from HU_0127_Cerebral‐Cortex_GSE140231 indicated that the DP1 receptor is mainly expressed in microglia in the cerebral cortex (Figure 2I). To determine the effect of aged mouse brain‐derived exosomes (A‐exo) on the expression of DP1 receptors in microglia, we stimulated BV2 microglia cultured in vitro with A‐exo. Flow cytometry results showed that the expression of DP1 receptors was significantly increased in the Con+A‐exo group compared with the Con+Y‐exo group (Figure 2J).

### 2.3 Brain‐Derived Exosomes From Aged Mice Induce Microglial DP1 Activation and Cellular Senescence

To investigate whether A‐exo directly acts on the DP1 receptor in microglia, we concurrently treated microglia with A‐exo and the DP1 receptor inhibitor asapiprant (abbreviated as asap in subsequent text and figures) to observe whether asap could reverse microglia activation, senescence‐associated secretory phenotype (SASP) secretion, and cellular senescence induced by A‐exo.

**FIGURE 3:**
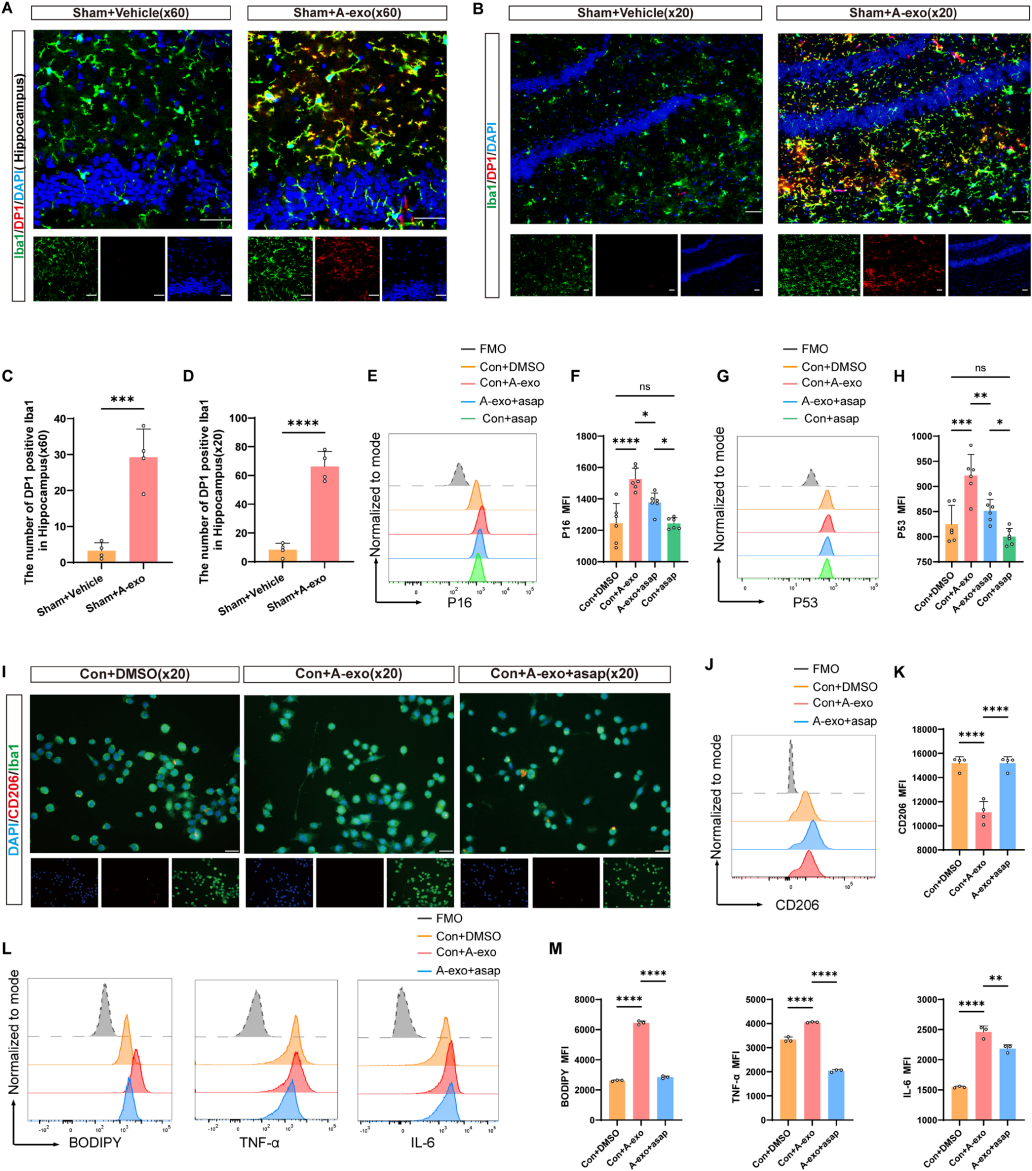
Brain‐derived exosomes from aged mice induce microglial DP1 activation and cellular senescence. (A) Representative immunofluorescence staining of DP1‐positive microglia. Red indicates DP1‐positive staining, Green indicates Iba1‐positive staining, and blue indicates positive DAPI nuclear staining. Scale bar = 20 μm. (C) Quantification of the number of DP1‐positive microglia between two groups (*n* = 4 mice per group). (B) Representative immunofluorescence staining of DP1‐positive microglia. Red indicates DP1‐positive staining, Green indicates Iba1‐positive staining, and blue indicates positive DAPI nuclear staining. Scale bar = 50 μm. (D) Quantification of the number of DP1‐positive microglia (*n* = 4 mice per group). (E) Flow cytometry analysis of P16 expression of microglia. (F) Quantification of P16. *n* = 6 per group. Data were representative of six independent experiments. (G) Flow cytometry analysis of P53 expression of microglia. (H) Quantification of P53. *n* = 6 per group. Data were representative of six independent experiments. (I) Representative immunofluorescence staining of CD206‐positive microglia. Red indicates CD206‐positive staining, Green indicates Iba1‐positive staining, and blue indicates positive DAPI nuclear staining. Scale bar = 50 μm. (J) Flow cytometry analysis of CD206 expression of microglia. (K) Quantification of CD206 expression of microglia. *n* = 4 per group. Data were representative of four independent experiments. (L) Flow cytometry analysis of BODIPY, TNF‐α and IL‐6 expression of microglia. (M) Quantification of BODIPY, TNF‐α and IL‐6. *n* = 3 per group. Data were representative of three independent experiments. (Data were represented as the mean ± SD. *p < 0.05, **p < 0.01, ***p < 0.001, and ****p < 0.0001.)

We injected A‐exo into the hippocampal region of the mouse brain and performed immunofluorescence staining on the hippocampal region of the mouse brain. Immunofluorescence analysis revealed a significant upregulation of DP1 receptor expression in microglia of the Sham+A‐exo group compared to the Sham+Vehicle group (Figure 3A–D). This indicates that A‐exo robustly activates the DP1 receptor on microglia.

We conducted interventions on BV2 microglia in vitro, grouping them as Con+DMSO, Con+A‐exo, A‐exo+asap, and Con+asap. Flow cytometry analysis revealed that A‐exo led to an increase in the senescence markers P16 and P53 in microglia. Compared to the A‐exo group, the expression of P16 and P53 in microglia was significantly reduced in the A‐exo+asap group. Additionally, asap had no effect on microglia (Figure 3E–H). Further immunofluorescence staining similarly found that A‐exo reduces the expression of CD206 in microglia. In comparison to the A‐exo group, the A‐exo+asap group promoted microglia to express more CD206, a marker of the anti‐inflammatory phenotype (Figure 3I). Similarly, flow cytometry analysis also showed that microglia treated with both A‐exo and asap exhibited increased expression of the anti‐inflammatory cytokine CD206, reduced lipid droplet accumulation, and decreased secretion of SASP factors such as TNF‐α and IL‐6 compared to microglia treated solely with A‐exo (Figure 3J–M). In summary, A‐exo leads to microglial DP1 activation and cellular senescence, and DP1 receptor inhibitors significantly ameliorate A‐exo‐induced microglial senescence and neuroinflammation.

### 2.4 DP1 Receptor Blockage Reverses A‐Exo Mediated Hyperactivation of Microglial and Peripheral Immune Cell Infiltration

**FIGURE 4:**
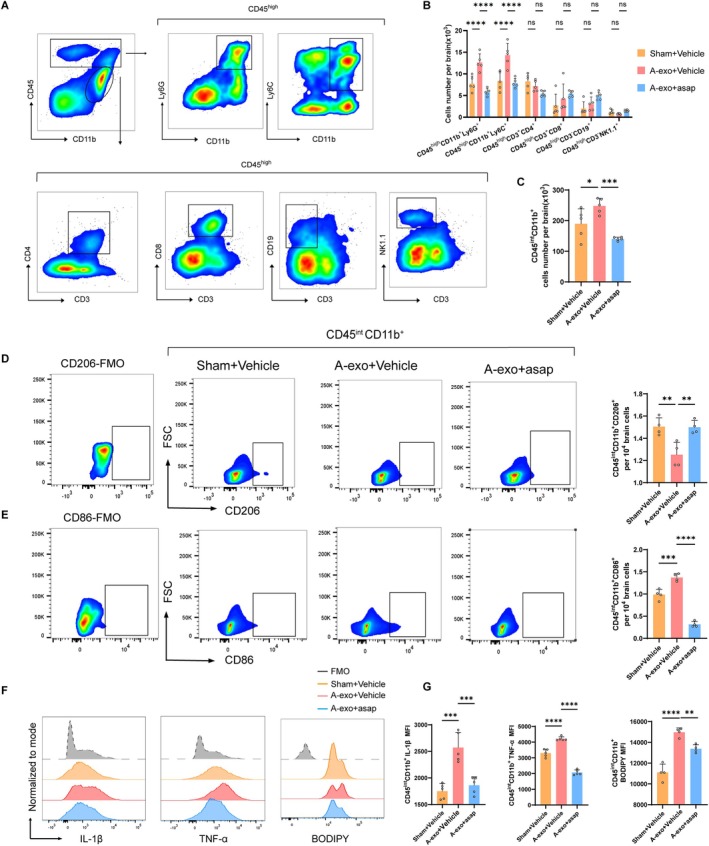
DP1 receptor blockage reverses A‐exo mediated hyperactivation of microglial and peripheral immune cell infiltration. (A) Gating strategy of brain‐infiltrating neutrophils (CD45^high^CD11b^+^Ly6G^+^), monocytes (CD45^high^CD11b^+^Ly6C^high^), CD4^+^ T cells (CD45^high^CD3^+^CD4^+^), CD8^+^ T cells (CD45^high^CD3^+^CD8^+^), B cells (CD45^high^CD3^−^CD19^+^) and NK cells (CD45^high^CD3^−^NK1.1^+^), as well as microglia (CD11b^+^CD45^int^). (B) Counts of brain‐infiltrating leukocyte subsets in the brains from indicated groups of mice (*n* = 5 mice per group). (C) Counts of brain‐infiltrating microglia (CD11b^+^CD45^int^). (D) Flow cytometry analysis of microglia (CD11b^+^CD45^int^) expressing CD206. Quantification of CD206. *n* = 4 mice per group. (E) Flow cytometry analysis of microglia (CD11b^+^CD45^int^) expressing CD86. Quantification of CD86. *n* = 4 mice per group. (F) Flow cytometry analysis of microglia (CD11b^+^CD45^int^) expressing inflammatory cytokine IL‐1β, TNF‐α, BODIPY. (G) Quantification of IL‐1β, TNF‐α, BODIPY. *n* = 5 mice per group. (Data were represented as the mean ± SD. *p < 0.05, **p < 0.01, ***p < 0.001, and ****p < 0.0001.)

To assess the influence of A‐exo on leukocyte infiltration into the brain. Using flow cytometry, we measured the counts of immune cell subsets in the brains of mice administered with A‐exo (Figure 4A). We found that, compared to normal mice, mice receiving A‐exo exhibited significantly increased numbers of microglia (CD45^int^ CD11b^+^), neutrophils (CD45^high^CD11b^+^Ly6G^+^), and monocytes (CD45^high^CD11b^+^Ly6C^high^) in their brains (Figure 4A–C). Conversely, the counts of other immune cell subsets, such as CD4^+^ T cells (CD45^high^CD3^+^CD4^+^), CD8^+^ T cells (CD45^high^CD3^+^CD8^+^), NK cells (CD45^high^CD3^−^NK1.1^+^), and B cells (CD45^high^CD3^−^CD19^+^), remained unchanged in the brains of A‐exo‐administered mice (Figure 4B). Notably, A‐exo increased the number of microglia expressing CD86, lipid droplets (BODIPY), IL‐1β and TNF‐α (Figure 4E–G), and decreased microglial expression of CD206 (Figure 4D). Brain tissue inflammatory cytokines were measured by enzyme‐linked immunosorbent assay (ELISA), and the results are presented in Figure S1C–E. The ELISA results from brain tissues were consistent with flow cytometry findings, demonstrating that A‐exo increased the expression of TNF‐α, IL‐6, and IL‐1β in the brain, thereby exacerbating neuroinflammation (Figure S1C–E). These results indicate that A‐exo promotes leukocyte infiltration and microglial inflammatory activity in the brains of normal young mice.

To investigate whether A‐exo activates microglia by acting on the DP1 receptor, we simultaneously intervened in mice with a DP1 receptor inhibitor (asap) and A‐exo. The results showed that, compared to the A‐exo+Vehicle group, the A‐exo+asap group significantly reduced the number of immune cells such as CD45^high^CD11b^+^Ly6G^+^ and CD45^high^CD11b^+^Ly6C^+^ in the brain (Figure 4B). Notably, the DP1 receptor inhibitor (asap) decreased the number of microglia expressing CD86, lipid droplets (BODIPY), IL‐1β and TNF‐α (Figure 4E–G), and increased microglial expression of CD206 (Figure 4D). These findings suggest that A‐exo activates microglia through the DP1 receptor, which in turn regulates the infiltration and activation of immune cells in the brain.

These results indicate that A‐exo induces the infiltration of immune cells such as CD45^high^CD11b^+^Ly6G^+^ and CD45^high^CD11b^+^Ly6C^+^ into the brain by activating the microglial DP1 receptor. A‐exo also stimulates microglia to express increased levels of CD86, IL‐1β, and TNF‐α through activation of the microglial DP1 receptor. DP1 receptor blockage reverses A‐exo‐mediated hyperactivation of microglia and leukocyte infiltration.

### 2.5 Microglial Depletion Alleviates Cognitive Decline and Cellular Senescence due to A‐Exo

**FIGURE 5:**
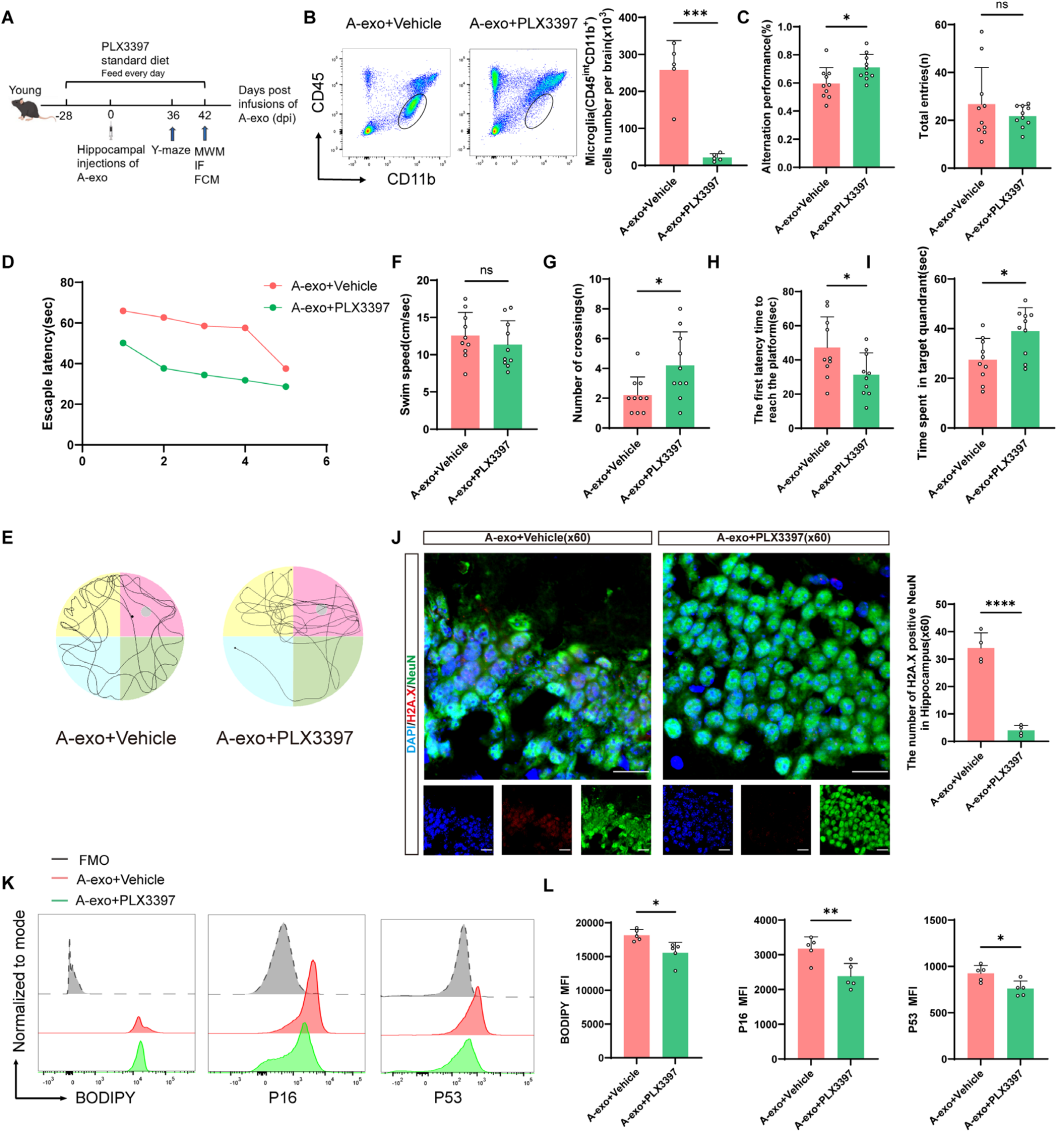
Microglial depletion alleviates cognitive decline and cellular senescence due to A‐exo. (A) The schematic diagram illustrates the experimental design of the animal part of the study. (B) (Left) Flow cytometry gating strategy of CD45^int^CD11b^+^(Microglia). (Right) Counts of microglia (CD11b^+^CD45^int^) *n* = 5 mice per group. (C) Y maze is performed on day 36 after exosomes injection into the hippocampus (*n* = 10 mice per group). (D) Escape latency in the training phase (*n* = 10 mice per group). (E) Representative traces in the Morris water maze. (F) Swim speed during the probe trial of MWM. (G) Number of crossings during the probe trial of MWM. (H) The first latency time to reach the platform during the probe trial of MWM. (I) Time spent in the target quadrant during the probe trial of MWM. (J) Representative immunofluorescence staining of H2A.X‐positive cells in the hippocampus. Red indicates H2A.X‐positive staining, green indicates NeuN‐positive staining, and blue indicates positive DAPI nuclear staining. Scale bar = 20 μm. (K) Flow cytometry analysis of BODIPY, P16, and P53 expression in mouse brain tissue. (L) Quantification of BODIPY, P16, and P53. *n* = 5 mice per group. (Data were represented as the mean ± SD. *p < 0.05, **p < 0.01, ***p < 0.001, and ****p < 0.0001.)

To further investigate whether A‐exo impairs cognitive function and induces cellular senescence in mice by acting on microglia, we first depleted microglia in the brain using the small‐molecule CSF1R inhibitor PLX3397 (Elmore et al. 2014). Microglia are the only immune cells in the central nervous system that express CSF‐1R under physiological conditions, and their development and survival depend on the CSF‐1R signaling pathway (Langmann et al. 2015; Waisman et al. 2015). Administration of a CSF‐1R inhibitor effectively eliminates microglia without adverse effects on other brain tissues. Mice were fed either a regular diet containing PLX3397 or a standard diet for 28 days, which was continued until the end of the experiment, to prevent subsequent microglial proliferation in the brain. The efficacy of PLX3397 in depleting microglia was confirmed by flow cytometry of brain tissue. Compared with mice fed the standard diet, the number of CD45^int^CD11b^+^ microglia in the brains of mice fed the PLX3397‐containing diet was significantly reduced (Figure 5B). Immunofluorescence analysis further confirmed robust microglial depletion following 28‐day PLX3397 intervention, as evidenced by quantitative assessment of Iba1^+^ cells (Figure S1F,G).

Y‐maze was performed at 36 days after A‐exo injection into the hippocampus to detect the cognitive function of mice. Compared to the A‐exo+PLX3397 group, the alternation performance of the A‐exo+Vehicle group significantly decreased, indicating impaired memory. In comparison to the A‐exo+Vehicle group, the A‐exo+PLX3397 group exhibited higher alternation performance. The above results showed that microglial depletion alleviates cognitive decline due to A‐exo (Figure 5C).

Between days 37 and 42 after A‐exo injection into the hippocampus, the water maze was performed. Mice in the A‐exo+Vehicle group exhibited impaired spatial learning and memory compared to the A‐exo+PLX3397 group. Treatment with PLX3397 significantly alleviates cognitive decline due to A‐exo, as evidenced by their swimming paths and learning curves (Figure 5D,E). During the training phase, mice in the A‐exo+Vehicle group exhibited significantly longer escape latencies compared to the A‐exo+PLX3397 mice (Figure 5D). In the probe test, the number of crossings over the target quadrant platform significantly decreased in the A‐exo+Vehicle group (Figure 5G), while the latency to first platform arrival increased (Figure 5H), indicating impaired spatial learning and memory function. Conversely, compared to the A‐exo group, mice in the A‐exo+PLX3397 group spent significantly more time in the target quadrant (Figure 5I) and had reduced latency to first platform arrival (Figure 5H), indicating recovery of spatial learning and memory function. Additionally, there were no significant differences in average swimming speed among the four groups of mice (Figure 5F). In conclusion, microglial depletion improves the effects of A‐exo on cognitive impairment.

Subsequently, we examined the effects of A‐exo treatment on cellular senescence in mice fed either the PLX3397‐containing diet or the standard diet. Compared with A‐exo treatment alone, the A‐exo+PLX3397 group has less expression of the DNA damage marker H2A.X (Figure 5J). This suggests that the A‐exo+PLX3397 treatment significantly ameliorated neuronal senescence. Flow cytometry results also indicated that, compared with the A‐exo+Vehicle group, the A‐exo+PLX3397 group exhibited a reduction in cellular lipid droplets, accompanied by a significant decrease in the expression of P16 and P53 (Figure 5K,L).

### 2.6 Microglial Depletion Suppresses Leukocyte Infiltration due to A‐Exo in Brain and Has No Significant Impact on Splenocytes

**FIGURE 6:**
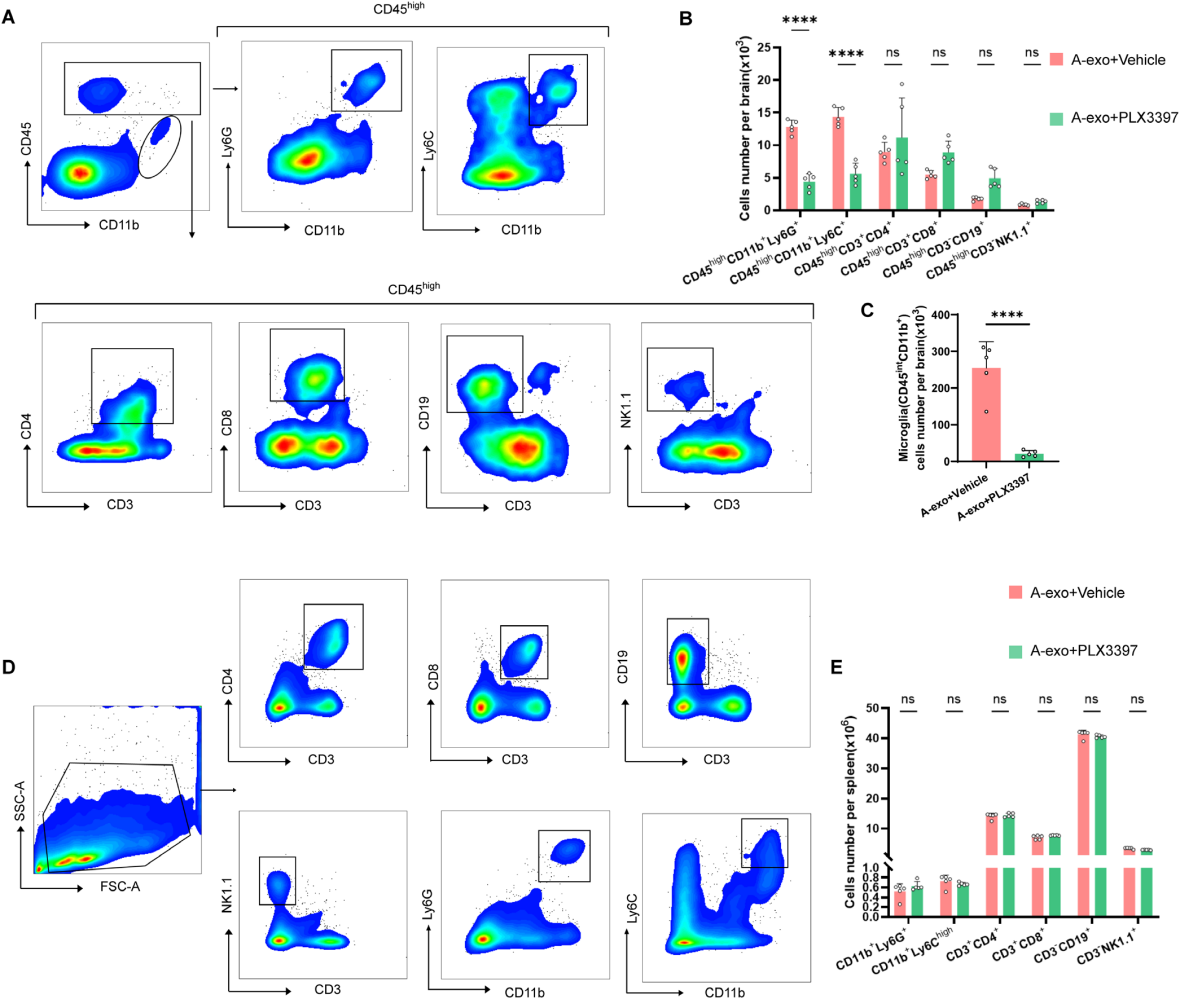
Microglial depletion suppresses leukocyte infiltration due to A‐exo in the brain and has no significant impact on splenocytes. (A) Gating strategy of brain infiltrating neutrophils (CD45^high^CD11b^+^Ly6G^+^), monocytes (CD45^high^CD11b^+^Ly6C^high^), CD4^+^ T cells (CD45^high^CD3^+^CD4^+^), CD8^+^ T cells (CD45^high^CD3^+^CD8^+^), B cells (CD45^high^CD3^−^CD19^+^) and NK cells (CD45^high^CD3^−^NK1.1^+^), as well as microglia (CD11b^+^CD45^int^). (B) Counts of brain‐infiltrating leukocyte subsets in the brains from indicated groups of mice. *n* = 5 mice per group. (C) Counts of brain‐infiltrating microglia (CD11b^+^CD45^int^). *n* = 5 mice per group. (D) Gating strategy of neutrophils (CD11b^+^Ly6G^+^), monocytes (CD11b^+^Ly6C^high^), NK cells (CD3^−^NK1.1^+^), CD4^+^ T cells (CD3^+^CD4^+^), CD8^+^ T cells (CD3^+^CD8^+^), and B cells (CD3^−^CD19^+^). (E) Cell counts of indicated immune cell subsets in spleens of mice. *n* = 5 mice per group. (Data are presented as mean ± SD. ****p < 0.0001.)

We next evaluated the impact of microglial depletion on A‐exo‐induced leukocyte infiltration in the brain. Using flow cytometry, we quantified immune cell subsets in the brains of mice subjected to hippocampal A‐exo injection with or without microglial depletion (Figure 6A). Compared to controls fed a standard diet, microglial depletion significantly reduced the counts of microglia (CD11b^+^CD45^int^), neutrophils (CD45^high^CD11b^+^Ly6G^+^), and monocytes (CD45^high^CD11b^+^Ly6C^high^) in A‐exo‐injected mice (Figure 6B,C). In contrast, other immune cell subsets—including CD4^+^ T cells (CD45^high^CD3^+^CD4^+^), CD8^+^ T cells (CD45^high^CD3^+^CD8^+^), NK cells (CD45^high^CD3^−^NK1.1^+^), and B cells (CD45^high^CD3^−^CD19^+^) remained unchanged following microglial depletion (Figure 6B).

We further investigated the impact of microglial depletion on splenic immune subsets in mice subjected to hippocampal A‐exo injection. Flow cytometry analysis revealed that the counts of splenic immune cells, including neutrophils (CD11b^+^Ly6G^+^), monocytes (CD11b^+^Ly6C^high^), NK cells (CD3^−^NK1.1^+^), CD4^+^ T cells (CD3^+^CD4^+^), CD8^+^ T cells (CD3^+^CD8^+^), and B cells (CD3^−^CD19^+^) remained comparable between the microglia‐depleted and control groups (Figure 6D,E). These results indicate that microglial depletion does not significantly alter splenic immune cell composition in A‐exo‐injected mice.

### 2.7 Brain‐Derived GFAP ^+^ Astrocytic Exosomes From Aged Mice Induce Pro‐Inflammatory Cytokine Secretion and Cellular Senescence in Microglia

**FIGURE 7:**
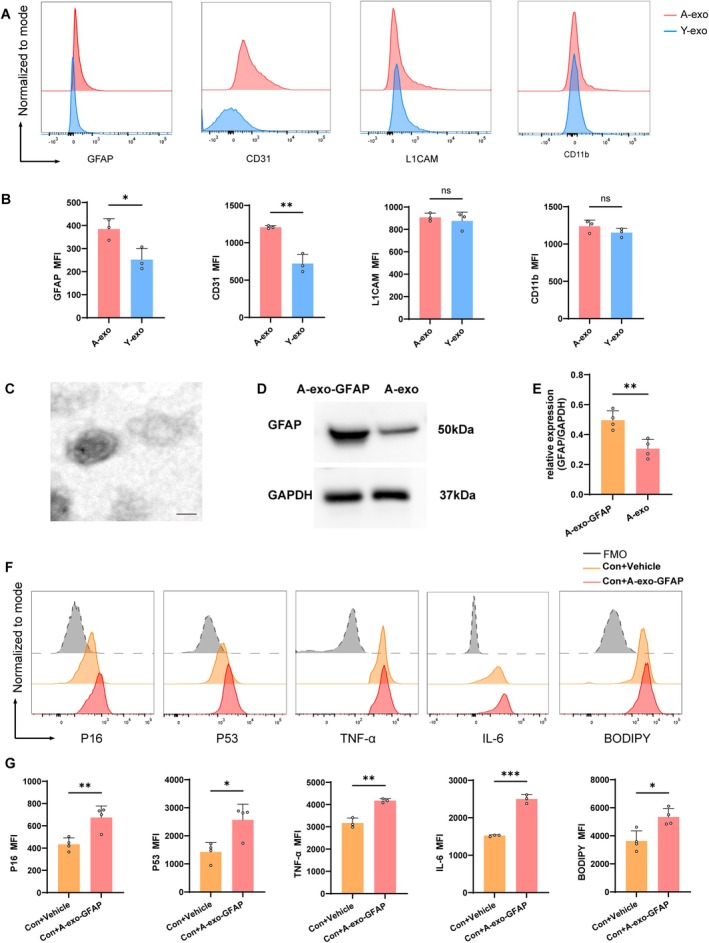
Brain‐derived GFAP+ astrocytic exosomes from aged mice induce pro‐inflammatory cytokine secretion and cellular senescence in microglia. (A) Flow cytometry analysis of GFAP, CD31, L1CAM, and CD11b expression in exosomes. (B) Quantification of GFAP, CD31, L1CAM, and CD11b. *n* = 3 per group. Data were representative of three independent experiments. (C) TEM image of purified exosomes from brain‐derived exosomes from aged mice. Scale bars, 100 nm. (D, E) Western blotting analysis of GFAP in the A‐exo‐GFAP and A‐exo. Protein levels were normalized to GAPDH (*n* = 4 mice per group). (F) Flow cytometry analysis of microglia expressing P16, P53, TNF‐α, IL‐6, and BODIPY. (G) Quantification of P16, P53, TNF‐α, IL‐6, and BODIPY. *n* = 4 mice per group. (Data were represented as the mean ± SD. *p < 0.05, **p < 0.01, and ***p < 0.001.)

To identify whether the cellular origins of A‐exo and Y‐exo are the same, we analyzed the exosomes using flow cytometry. We found that A‐exo exhibited higher expression of GFAP and CD31 compared with Y‐exo, while there were no significant differences in the expression of L1CAM and CD11b between A‐exo and Y‐exo (Figure 7A,B). These results suggest that A‐exo may primarily originate from astrocytes and endothelial cells in the aged brain.

Compared with Y‐exo, A‐exo exhibited significantly higher expression of PTGDS, which is predominantly expressed in astrocytes. Based on this observation, we further investigated the effects of brain‐derived astrocytic exosomes from aged mice on microglia. We isolated astrocytic exosomes with high GFAP expression (A‐exo‐GFAP) from brain‐derived exosomes of aged mice and used them to intervene in BV2 microglia cultured in vitro. TEM revealed that A‐exo‐GFAP displayed intact exosomal morphology (Figure 7C). Western blot (WB) analysis showed that A‐exo‐GFAP had significantly higher GFAP expression compared to A‐exo (Figure 7D,E). Flow cytometry further demonstrated that A‐exo‐GFAP increased the expression of P16, P53, TNF‐α, IL‐6, and BODIPY in microglia (Figure 7F,G). Collectively, these findings indicate that astrocytic exosomes (A‐exo‐GFAP) from the brains of aged mice exacerbate neuroinflammation and induce microglial senescence.

### 2.8 DP1 Receptor Blockage Ameliorates Cellular Senescence and Cognitive Decline in Aged Mice

To investigate whether A‐exo induces microglial senescence in aged mice, we injected A‐exo into the hippocampus of aged mice (18 months old) and examined microglial senescence in aged mice. The results, presented in Figure S2, show that compared with young mice, microglia in the brains of aged mice exhibited a senescent phenotype, characterized by increased expression of P16, P53, and lipid droplets. A‐exo further enhanced microglial senescence in the brains of aged mice (Figure S2A,B).

**FIGURE 8:**
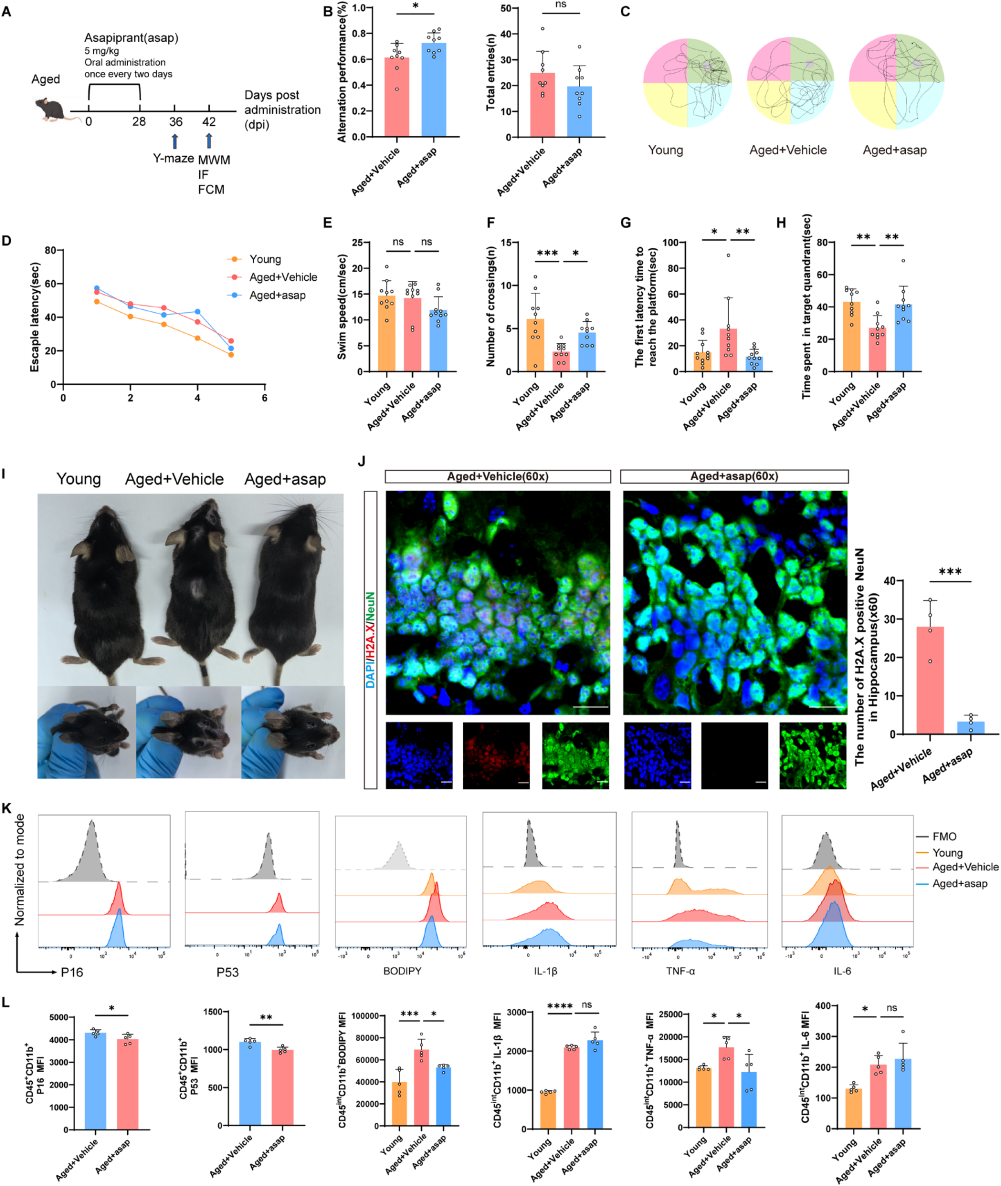
DP1 receptor blockage ameliorates cellular senescence and cognitive decline in aged mice. (A) The schematic diagram illustrates the experimental design of the animal part of the study. (B) Y maze is performed on day 36 after exosomes injection into the hippocampus (*n* = 9 mice per group). (C) Representative traces in the Morris water maze. (D) Escape latency in the training phase (*n* = 10 mice per group). (E) Swim speed during the probe trial of MWM. (F) Number of crossings during the probe trial of MWM. (G) The first latency time to reach the platform during the probe trial of MWM. (H) Time spent in the target quadrant during the probe trial of MWM. (I) Representative images of mouse body and facial hair color and shedding. (J) Representative immunofluorescence staining of H2A.X‐positive cells in the hippocampus. Red indicates H2A.X‐positive staining, green indicates NeuN‐positive staining, and blue indicates positive DAPI nuclear staining. Scale bar = 20 μm. (K) Flow cytometry analysis of microglia (CD11b^+^CD45^int^) expressing P16, P53, BODIPY, IL‐1β, TNF‐α and IL‐6. (L) Quantification of P16, P53, BODIPY, IL‐1β，TNF‐α and IL‐6. *n* = 5 mice per group. (Data were represented as the mean ± SD. *p < 0.05, **p < 0.01, ***p < 0.001, and ****p < 0.0001.)

To investigate the impact of the DP1 receptor inhibitor (asap) on cognitive function in aged mice, we administered the DP1 receptor inhibitor (asap) via gavage to 18‐month‐old mice. We assessed the Y‐maze performance of mice at 36 days and evaluated the effect of asap on recognition memory in aged mice by calculating alternation performance. Compared to the Young group, the alternation performance of the Aged+Vehicle group significantly decreased, indicating impaired memory. In comparison to the Aged+Vehicle group, the Aged+asap group exhibited higher alternation performance. This suggests that asap treatment improves cognitive decline in aged mice (Figure 8B).

Between days 37 and 42 after orally administering DP1 receptor inhibitor Asapiprant, a water maze was performed. Mice in the Aged+Vehicle group exhibited impaired spatial learning and memory compared to the Young group. Treatment with asap significantly improved the spatial learning ability of the aged mice, as evidenced by their swimming paths and learning curves (Figure 8C,D). During the training phase, the Aged+ Vehicle mice of the group showed a decrease in escape latency, while mice in the Aged+Vehicle group exhibited significantly longer escape latencies compared to the Young mice. In contrast, mice treated with asap showed shorter escape latencies compared to the Aged+Vehicle counterparts (Figure 8D). In the probe test, the number of crossings over the target quadrant platform significantly decreased in the Aged+Vehicle group (Figure 8F), while the latency to first platform arrival increased (Figure 8G), indicating impaired spatial learning and memory function. Conversely, compared to the vehicle‐treated counterparts, mice in the Aged+asap group spent significantly more time in the target quadrant (Figure 8H) and had reduced latency to first platform arrival (Figure 8G), indicating recovery of spatial learning and memory function. Additionally, there were no significant differences in average swimming speed among the four groups of mice (Figure 8E). In conclusion, the aged mice showed impaired spatial learning and memory function compared to the Young mice, while treatment with asap significantly alleviated cognitive decline in aged mice.

To our amazement, we also found that the DP1 receptor inhibitor significantly improved hair loss in aged mice (Figure 8I). Flow cytometry results further indicated that the DP1 receptor inhibitor markedly reduced the expression of P16 and P53 in microglia within the brains of aged mice (Figure 8K,L). Immunofluorescence staining was conducted, and the results showed that compared to aged mice, asap treatment significantly decreased the expression of the neuronal DNA damage marker (H2A.X) (Figure 8J), improving cellular senescence in aged mice. Additionally, flow cytometry results demonstrated that the DP1 receptor inhibitor significantly decreased lipid droplet accumulation (BODIPY) in microglia and reduced the expression of senescence‐associated secretory phenotype (SASP) cytokines, including IL‐1β, TNF‐α, and IL‐6, in microglia within the brains of aged mice (Figure 8K,L), thereby ameliorating neuroinflammation. These findings suggest that DP1 receptor blockage ameliorates cellular senescence and cognitive decline in aged mice.

## 3 Discussion

Exosomes in the aging brain contribute to brain senescence and cognitive decline, yet a blanket inhibition of their secretion is impractical. This is because exosome secretion from the aging brain serves as a crucial mechanism for waste clearance and intercellular communication. The aim of this study is to elucidate the specific mechanisms underlying the cognitive‐impairing effects of senescent brain‐derived exosomes, with the objective of mitigating cellular senescence and cognitive decline by targeting their detrimental aspects.

This study demonstrates that brain‐derived exosomes from aged mice (A‐exo) drive cognitive decline by activating the PTGDS/PGD2/DP1 signaling axis in microglia. Key findings include: (1) A‐exo administration induces microglial overactivation, lipid droplet accumulation, and senescence‐associated secretory phenotype (SASP) secretion, leading to spatial memory deficits in young mice. (2) Proteomic analysis identifies PTGDS as a highly enriched protein in A‐exo, which elevates central and peripheral PGD2 levels, thereby activating DP1 receptors on microglia. (3) DP1 receptor activation triggers myeloid cell infiltration, neuroinflammation, and cellular senescence, which are reversed by microglial depletion or pharmacological DP1 inhibition (asapiprant). (4) Critically, DP1 blockade ameliorates cognitive decline, reduces neuronal DNA damage, and mitigates SASP secretion in aged mice, highlighting its therapeutic potential.

This work provides several novel insights. First, it is the first to establish that aged brain‐derived exosomes act as systemic mediators of neuroinflammation and cognitive decline via the PTGDS/PGD2/DP1 pathway—a mechanism distinct from canonical cytokine‐driven inflammation (H. Zhang, Lin, et al. 2021). Second, the identification of DP1 as a key regulator of microglial senescence and SASP secretion bridges the gap between aging‐associated exosomal cargo and neurodegenerative outcomes (Ji et al. 2024). Third, the therapeutic efficacy of asapiprant in reversing age‐related cognitive deficits offers a repurposed drug candidate for neuroprotection, a strategy underexplored in aging research.

Despite the elusive nature of the underlying mechanisms of aging, studies have shown that transfusing blood from young mice into old mice can reverse age‐related cognitive decline. Injection of serum exosomes from aged rats into aged ischemic rats results in microglial dysfunction, exacerbating synaptic dysfunction and sensorimotor deficits (Garcia‐Martin et al. 2021). Ruckh et al. found that a youthful systemic environment can enhance myelin regeneration in aged animals, and exosomes produced in the brain microenvironment of young animals mimic this myelin‐promoting effect (Goulielmaki et al. 2020). Although these studies have demonstrated therapeutic potential for cellular senescence and age‐related diseases, the specific mechanisms of action remain unclear. Exosomes, as important mediators of intercellular communication, may play a pivotal role in these processes. Exosomes in the aging brain environment contain higher levels of contents related to DNA and mitochondrial damage, immune inflammation, and ROS accumulation. Senescent brain‐derived exosomes release a plethora of inflammatory factors generated during brain aging, which can alter the intracellular environment and amplify the senescent microenvironment, transmitting senescence signals or patterns to surrounding cells or tissues (Squillace and Salvemini 2022). Intriguingly, a recent study revealed that extracellular vehicles (EVs) from the blood of patients with major depressive disorder induce depressive‐like behaviors in wild‐type mice, whereas injection of EVs derived from the blood of healthy individuals alleviates depressive‐like behaviors in mouse models of depression (C. Wang, Börger, et al. 2022). This study emphasizes the unexplored role of exosomes as mediators of cognitive and behavioral changes.

Prior studies have implicated exosomes in propagating neuroinflammation, but their age‐dependent roles remain poorly defined. For instance, plasma exosomes from Alzheimer's patients carry proinflammatory miRNAs (L. Wang, Zhen, et al. 2022), yet our work uniquely links aging‐specific exosomal PTGDS to DP1‐mediated microglial dysfunction. Similarly, while microglial senescence is recognized as a driver of neurodegeneration (Kim et al. 2025), this study uncovers a novel exosome‐microglia crosstalk mechanism. The role of DP1 in neuroinflammation has been suggested in models of multiple sclerosis (Verma et al. 2021), but its involvement in aging and cognitive decline is unprecedented. Notably, our findings align with reports that prostaglandin signaling exacerbates neuroinflammation (Stark and Penn 2024), yet contrast with studies emphasizing neuroprotective roles of PGD2 in acute injury (I. Mohri et al. 2006), underscoring context‐dependent effects. Finally, the efficacy of CSF1R inhibition in attenuating microglial activation echoes work by Spangenberg et al. (E. E. Spangenberg et al. 2016), but our data extend this by linking microglial depletion to reduced peripheral immune infiltration via DP1 modulation.

Prostaglandin D2 Receptor 1 (DP1) is a key mediator in immunosenescence, inflammatory modulation, neuroprotection, and neurodegenerative diseases. The prostaglandin D2 receptor 1 (DP1), a primary receptor for prostaglandin D2 (PGD2), is widely distributed in the central nervous system (CNS) and plays a pivotal role in regulating diverse physiological and pathological processes.

The DP1 signaling pathway becomes hyperactive with aging, leading to impaired antigen‐presenting efficiency in dendritic cells and excessive neutrophil infiltration into infected tissues, which exacerbates destructive inflammatory responses (D. Sang et al. 2023; Trotta et al. 2025; Wong et al. 2022). Pharmacological intervention using the PTGDR antagonist BGE‐175 (asapiprant) effectively blocks PGD2‐DP1 signaling, thereby rejuvenating immune function in aged individuals. Experimental studies demonstrated that BGE‐175 treatment increased the survival rate of aged COVID‐19‐infected mice from 0% to 90%, concurrently reducing pulmonary inflammation (Wong et al. 2022). Notably, BGE‐175 targets host immunity rather than viral replication, suggesting broad therapeutic potential against multiple infections. A Phase II clinical trial is currently evaluating its efficacy in elderly COVID‐19 patients.

Chronic sleep deprivation induces cerebral PGD2 accumulation, which is transported peripherally via the ABCC4 transporter, resulting in DP1 receptor activation, neutrophilia, and cytokine storm‐like syndromes (D. Sang et al. 2023). Genetic ablation of PGD2 synthase (PTGDS) or DP1 receptors in mice significantly attenuated inflammatory responses and improved survival rates following sleep deprivation, highlighting the central role of the PGD2/DP1 axis in sleep‐immune crosstalk. These findings suggest that DP1 antagonists may represent a novel strategy for managing immune hyperactivation associated with sleep disorders.

Emerging evidence implicates prostaglandin‐mediated signaling in retinal cells as a driver of inflammatory responses linked to early diabetic retinopathy. Specifically, PGD2‐DP1 interactions promote microvascular dysfunction and leukocyte adhesion, exacerbating retinal pathology (Stark and Penn 2024). In AD models, DP1 antagonism reduces cerebral β‐amyloid (Aβ) deposition and ameliorates cognitive deficits, likely through modulating microglial function to enhance Aβ clearance and suppress neuroinflammation (Stark and Penn 2024). Further mechanistic studies reveal that PGD2‐mediated crosstalk between microglia and astrocytes amplifies astrogliosis and demyelination, as observed in the twitcher mouse model of leukodystrophy (I. Mohri et al. 2006).

In the brain, DP1 receptors are predominantly expressed in microglia and excitotoxic neurons. During excitotoxic hippocampal injury, the abundant production of PGD2 and PGD2‐induced microglial activation triggers neuroinflammation and further neurodegenerative changes (Wallace et al. 2022). The increase in PGD2 and microglial activation is closely related to neuroinflammation and neurodegeneration in neurological deficits (D. Sang et al. 2023). This aligns with our findings, where exosomes derived from the brains of aged mice (A‐exo) cause cognitive decline in normal young mice, induce microglial overactivation, lipid droplet accumulation, and senescence‐associated secretory phenotype (SASP) secretion. This abnormal microglial activity is due to the high expression of PTGDS in A‐exo following mouse senescence, leading to increased central and peripheral DP1 ligand PGD2 levels and subsequent sustained DP1 signaling activation. Consequently, this results in increased myeloid cell adhesion, cellular senescence, and cognitive decline through the generation of senescent, pro‐inflammatory microglia. Blocking the DP1 receptor can ameliorate A‐exo‐mediated microglial overactivation, myeloid cell infiltration, and cellular senescence.

Several limitations warrant consideration. Although we observed an increase in the DP1 ligand PGD2 in both the brain and peripheral blood of aged mice, the clinical relevance of this finding to age‐related cognitive decline and neurodegenerative diseases awaits further investigation in a large cohort of aging individuals.

Future studies should prioritize: (1) Translational validation: Investigating PTGDS/PGD2/DP1 axis alterations in human aging and neurodegenerative cohorts, leveraging cerebrospinal fluid or plasma exosomes (A. G. Thompson et al. 2016). (2) Mechanistic expansion: Exploring DP1 signaling downstream effectors using single‐cell transcriptomics (Hammond et al. 2019). (3) Therapeutic optimization: Developing brain‐penetrant DP1 inhibitors or exosome‐targeted therapies to enhance specificity (Dumontet et al. 2023). These efforts will advance our understanding of age‐related neuroinflammation and accelerate clinical translation.

In summary, this study found that brain‐derived exosomes from aged mice induce sustained activation of microglial DP1 signaling. The continuous increase of PGD2 ligands in both the circulation and central nervous system results in a proinflammatory microglial phenotype, thereby driving the progression of neuroinflammation after aging. This process can be targeted therapeutically with DP1 receptor inhibitors. Our findings provide a theoretical basis for improving aging and age‐related neuroinflammation and cognitive decline. DP1 receptor inhibitors have the potential to prevent age‐related neurofunctional impairments and cognitive decline, making them promising therapeutic targets for treating age‐related neurological diseases. Our results uncover the underlying mechanism by which microglial activation induces cognitive decline after brain aging and identify modifiable processes that may serve as potential therapeutic targets.

## 4 Methods

### 4.1 Animals

In this research, specific‐pathogen‐free (SPF) elderly male C57BL/6J mice (aged 18–20 months, weighing 30–35 g) and young male C57BL/6J mice (6–8 weeks old, weighing 20–22 g) were obtained from Beijing Vitalstar Biotechnology Co. Ltd. All the mice were kept in the Animal Center of Tianjin Medical University under SPF conditions. The environment featured a 12‐h light/dark cycle, a temperature of 22°C ± 2°C, and a humidity of 45%. They had free access to standard rodent feed and sterile water. Experimental animal operations were carried out following the protocols approved by the Animal Care and Use Committee of Tianjin Medical University (Tianjin, China), in accordance with the NIH Guidelines for the Care and Use of Laboratory Animals. All procedures were approved by the Animal Care and Use Committee of Tianjin Medical University General Hospital (Approval No. IRB2022‐DWFL‐074) and strictly conformed to the ARRIVE guidelines.

### 4.2 Isolation of Brain Exosomes

Brain tissues from either 18‐ to 20‐month‐old or 6‐ to 8‐week‐old wild‐type mice, which were finely minced using a sterile scalpel and placed into a 15 mL conical tube containing 3 mL of Hibernate A solution (A1247501, Thermo Fisher Scientific) with 20 units/mL of papain (Worthington) for enzymatic digestion at 37°C for 20 min (Bodart‐Santos et al. 2023). The enzymatic digestion process was halted by adding 7 mL of ice‐cold Hibernate A solution, which was supplemented with protease and phosphatase inhibitors. The tissue suspension was gently pipetted approximately 30 times using a 10 mL plastic pipette to ensure dissociation and homogeneity. The suspension was then subjected to sequential centrifugation steps at 300 × g for 10 min, 2000 × g for 10 min, and 10,000 × g for 30 min at 4°C to remove cells and debris of varying membrane sizes. The resulting supernatant was filtered through a 0.2 μm syringe filter and centrifuged at 100,000 × g for 70 min at 4°C using a SW41Ti Beckman rotor. The pellet containing exosomes was resuspended in 0.5 mL of PBS (Bodart‐Santos et al. 2023).

### 4.3 Nanoparticle Tracking Analysis of Exosomes

The particle size distribution of brain‐derived exosomes was assessed using NanoSight NS300 (Malvern Instruments, Worcestershire, UK) and ZetaView PMX 110 V3.0 (Particle Metrix, Meerbusch, Germany). For both instruments, exosomes were diluted in PBS. NanoSight results were averaged from three 1‐min readings per sample (Lei et al. 2021). ZetaView analysis involved two cycles, examining 11 cell positions with pre‐acquisition settings of sensitivity 85, frame rate 30 fps, and shutter/laser pulse at 55. Post‐acquisition settings were set to a minimum brightness of 20, with particle sizes ranging from 5 to 500 pixels (Bodart‐Santos et al. 2023).

### 4.4 Transmission Electron Microscopy

Brain‐derived exosome suspensions were fixed with 4% paraformaldehyde (diluted 1:1 to 2% final concentration) at 4°C overnight and stored at −20°C. After thawing, they were vortexed and centrifuged for 30 s. Exosomes were adsorbed onto Formvar‐coated Ni mesh grids by placing 5‐μL drops on the grids for 20 min in a dry environment. Negative staining was done using 100 μL of 1% aurothioglucose or 2% uranyl acetate, followed by excess removal with lens paper. The grids were then examined under Tecnai Spirit or Talos L120C transmission electron microscopes at 120 kV. Electron micrographs were captured at magnifications ranging from 8500× to 17,500× (Bodart‐Santos et al. 2023).

### 4.5 Immunofluorescence Staining

The cerebral tissue was initially fixed in phosphate‐buffered saline (PBS) containing 4% paraformaldehyde (PFA) for a duration of 12 h. This was followed by immersion in PBS with 15% sucrose at 4°C for 24 h, and subsequently in PBS with 30% sucrose at 4°C for 48 h. Once the tissue had settled, it was embedded in OCT compound and sliced into 10 μm sections using a cryostat. The sections were then exposed to 0.3% Triton X‐100 for 30 min at ambient temperature (Ge et al. 2020; Santos‐Garcia et al. 2023). Following this, the sections were blocked with PBS containing 10% goat serum for 1 h at room temperature and incubated overnight at 4°C with primary antibodies targeting CD206 (1:200, Santa Cruz Biotechnology), H2A.X (1:500, CST), NeuN (1:500, Abcam), and Iba1 (1:500, Abcam). After thorough washing with PBS, the sections were treated with secondary antibodies for 1 h (Alegre‐Zurano et al. 2023). The cell nuclei were counterstained with DAPI (S2110, Solarbio) (Q. Yang et al. 2020). Co‐localization was quantified using ImageJ Coloc2 and Pearson correlation coefficient (*r*) values. Imaging was performed using a Leica confocal microscope TCS SP8 or Nikon C2, and analysis was conducted using ImageJ software. ImageJ Coloc2 was used for quantifying co‐localization, and Pearson's correlation coefficient (*r*) values were calculated to assess the correlation between co‐localized voxels in different color channels.

### 4.6 Proteomic Analysis

ECHO Biotech conducted proteomic analysis on exosomes isolated from the brain tissues of young and aged rats. Protein concentrations of each exosome were measured using a Qubit Fluorometer (Invitrogen), and each sample (10 μg) was processed via 10% SDS‐PAGE. The gel bands were excised and processed for in‐gel trypsin digestion utilizing a ProGest robotic system (DigiLab). Comparative analysis of proteomic profiles between Y‐exo and A‐exo was conducted using nano‐liquid chromatography coupled with tandem mass spectrometry (nanoLC–MS/MS). This was performed with a Waters Nano Acquity HPLC system connected to a Thermo Fisher quadrupole‐orbitrap high‐resolution mass spectrometer. The acquired LC–MS/MS raw data were analyzed using Mascot (Matrix Science) and queried against the UniProt Rat database. The Mascot Dial‐A‐Truck (DAT) files were subsequently imported into Scaffold (Proteome Software) for validation, filtering, and compilation of a non‐redundant protein list for each sample. The data were refined based on a 1% false discovery rate (FDR) for both proteins and peptides, with a requirement of at least 2 unique peptides per protein. Protein identifications were considered valid if they were identified by a minimum of 2 peptides or a single unique peptide with an FDR below 1%.

### 4.7 Biological Functions and Pathway Analysis

Functional annotation of the identified proteins was performed using Blastp (Blast2GO version 4) and cross‐referenced with the Gene Ontology database (accessible at http://geneontology.org). To categorize proteins based on their similarity, hierarchical clustering was applied, utilizing the Pheatmap package (available at https://CRAN.R‐project.org/package=pheatmap, Estonia). This method organizes data into clusters according to their similarity matrix. For the visualization of significant changes across large datasets, a volcano plot was generated using the Ggplot2 package (https://ggplot2.tidyverse.org, New Zealand), which helps in identifying variations in data points among replicates. The analysis used mouse proteins as a reference for determining enrichment values. The functional enrichment of differentially expressed exosomal proteins was statistically assessed using Fisher's exact test within Blast2GO. Differentially expressed proteins were considered significant with an absolute fold change ≥ 1.5 and a corrected *p*‐value < 0.05. PPI networks were obtained with the STRING database and visualized using Cytoscape 3.9.1 (Y. Zhang et al. 2023). Gene Set Enrichment Analysis (GSEA) (Subramanian et al. 2005) was performed using R package “clusterProfiler” (Yu et al. 2012) and “GSEABase”, The DISCO databasethe, ScAgeAnno database, and HUSCH database help analyze the functionality of PTGDS.

### 4.8 Stereotaxic Infusion of Brain‐Derived Exosomes Into Mouse Hippocampus

Male wild‐type (WT) mice aged 6 to 8 weeks received intracranial infusions of brain‐derived exosomes from aged mice (18–20 months). The mice were anesthetized with 5% isoflurane (Cristalia) for 3 to 5 min and maintained at 2.5% isoflurane using a vaporizer system during the injection process. The exosomes (2.3 μg in 3 μL) were bilaterally injected into the hippocampus at coordinates (−2.0 AP, ±1.3 ML, −2.2 DV) using stereotaxic surgery. The mice were euthanized 42 days post‐infusion.

### 4.9 Drug Treatment

Asapiprant (asap) is a potent and selective DP1 receptor antagonist. Asapiprant (in 50% DMSO) at a dosage of 5 mg/kg was administered starting 2 h post‐exosome injection into the hippocampus of mice, once every 2 days for a total of 28 days. The vehicle control group received an equivalent volume orally (Sang et al. 2023).

Aged male C57BL/6J mice (19 months old, weighing 30–35 g) were treated with asapiprant at a dose of 5 mg/kg (in 50% DMSO) every 2 days for a total of 28 days. The vehicle control group received an equal amount orally.

### 4.10 Enzyme‐Linked Immunosorbent Assay

Serum and brain PGD2 concentrations were determined using a mouse PGD2 quantitative enzyme‐linked immunoassay kit (MLBIO, YJ911182) (Iwasa et al. 2021). Obtain serum samples and brain tissue samples and follow the instructions of the ELISA kit.

### 4.11 Morris Water Maze

The Morris water maze (MWM) test was utilized to evaluate the memory and spatial learning capabilities of murine subjects (Baeske et al. 2024). All mice underwent a training regimen aimed at locating a submerged platform within an opaque aquatic environment. For the initial 5 days of training, the platform (with a diameter of 10 cm) was submerged 1 cm beneath the water surface. Each mouse participated in four trials daily, each lasting 90 s, with a consistent starting quadrant and trial sequence for individual trials but varied among mice to prevent bias. The primary outcome measure was escape latency, defined as the time taken to discover the submerged platform, serving as an indicator of memory and spatial learning proficiency (capped at 90 s). Mice that did not locate the platform within this period were gently guided to it (Katz et al. 2015). Once the platform was found, the mice were allowed to stay on it for 15 s. On the sixth day, a spatial probe test (SPT) was performed by removing the platform and allowing each mouse to swim freely for 90 s. The Stoelting video tracking system was then used to measure various parameters, such as the number of times the mice crossed the platform's previous location, the time taken to first reach that location, and the swimming speed (Xiao‐Hang et al. 2024).

### 4.12 Y‐Maze Test

The Y‐maze apparatus is employed as a tool to evaluate short‐term learning and memory functions in murine models. Constructed from durable blue opaque polyethylene plastic, the Y‐maze comprises three arms designated as A, B, and C. During the procedure, mice are positioned at the central nexus of the three arms and granted unrestricted access to explore the maze for a duration of 8 min (Z. J. Yang et al. 2024). Spontaneous alternation behavior is recorded when an animal sequentially enters three distinct arms (e.g., ABC, CAB, ACB, BCA, CBA, BAC). The percentage of spontaneous alternations (%) is calculated using the formula: (number of spontaneous alternations/[total number of arm entries minus 2]) × 100. This metric serves as a quantitative indicator of short‐term memory and learning efficiency in the mice (Lee et al. 2024).

### 4.13 Microglial Depletion

To achieve in vivo microglial depletion, mice were administered a standard diet enriched with PLX3397 (Catalog No. HY‐16749; MedChemExpress Inc.), a CSF1R (colony‐stimulating factor 1 receptor) inhibitor (X. Li, Li, Jin, et al. 2023). This dietary regimen was initiated at least 28 days before the intrahippocampal administration of exosomes to ensure optimal microglial elimination, which was sustained throughout the duration of the study (Chauquet et al. 2024).

### 4.14 Cell Culture and Drug Treatment In Vitro

BV2 microglia were sourced from the Chinese Academy of Sciences Cell Resource Center for Basic Research (Beijing, China) and cultured in DMEM/F12 medium (Thermo Fisher Scientific) enriched with 10% ex‐fetal bovine serum (FBS), 100 U/mL penicillin, and 100 mg/mL streptomycin. The cells were incubated in a humidified environment with 5% CO_2_ at 37°C (Yin et al. 2020). Asapiprant (asap), a selective and potent DP1 receptor antagonist, was used at a concentration of 10 μM (D. Sang et al. 2023). BV2 cells were pretreated with 10 μM asap for 1 h, and then were intervened with exosomes at a concentration of 80 μg/mL for 24 h before detecting relevant indicators.

### 4.15 Flow Cytometry Analysis

Flow cytometry was employed to measure immune cell populations and evaluate cytokine production in the brain, following established protocols (Cheng et al. 2024). Between 42 and 45 days after the injection of exosomes into the mouse hippocampus, the brains were perfused with PBS, harvested, and treated with collagenase IV to create a single‐cell suspension. Myelin was removed using a 30% Percoll solution, and the resulting cell pellet was resuspended in a 1% BSA solution for staining. All antibodies were purchased from Biolegend (San Diego, CA, USA), unless otherwise specified. The antibodies used in this study included: CD45 (30‐F11), CD11b (M1/70), Ly6G (1A8), F4/80 (BM8), Ly6C (HK1.4), CD3 (17A2), CD4 (GK1.5), CD8 (53–6.7), NK1.1 (PK136), CD19 (6D5), tumor necrosis factor alpha (TNF‐α) (MP6‐XT22), IL‐1β (NJTEN3), IL‐6 (48‐7061‐82) (Cheng et al. 2024). P16(sc‐1661) and P53(sc‐126) were sourced from Santa. DP1(56773) was sourced from SAB. BODIPY (GC42959) was sourced from Glpbio (USA) (Guo et al. 2023). Flow cytometry was performed using a FACS Aria flow cytometer, and the data were analyzed with FlowJo 10.6.2 software.

### 4.16 Western Blotting

Western blotting was performed as previously described (Li et al. 2025). For protein extraction, brain tissues from each experimental group were lysed using a lysis buffer containing protease and phosphatase inhibitors. Brain tissues were homogenized to extract proteins into the supernatant fraction, followed by protein quantification using the bicinchoninic acid (BCA) assay. Specific primary antibodies were applied to polyvinylidene fluoride (PVDF) membranes and incubated overnight at 4°C. All antibodies were diluted at a 1:1000 ratio, except GAPDH, which was prepared at a 1:10,000 dilution. The next day, membranes underwent three rounds of washing to remove unbound antibodies. Subsequently, membranes were incubated with secondary antibodies (Zhongshan Golden Bridge, Beijing, China) diluted at 1:5000 for 1 h at room temperature. Immunoblots were developed using an enhanced chemiluminescence detection system and visualized with a ChemiDoc Touch Imaging System (Bio‐Rad, Hercules, CA, USA). Densitometric analysis of protein band intensity was performed using ImageJ software (National Institutes of Health, Version 1.53), providing a quantitative assessment of protein expression levels.

### 4.17 Isolation of Astrocytes Exosomes From Aged Brain

To determine the effects of brain‐derived astrocytic exosomes from aged mice on microglia, we collected GFAP‐positive A‐exo (A‐exo‐GFAP) and performed in vitro interventions on microglia. GFAP^+^ exosomes were isolated from total exosomes using a co‐immunoprecipitation assay (L. Li, Li, Bai, et al. 2023). Briefly, GFAP antibody (Cat. No. 60190‐1‐lg, Proteintech) was incubated with Protein G magnetic beads (Cat. No. 10007D; Thermo Fisher Scientific) at room temperature for 1 h. The isolated total exosomes were then incubated with antibody‐coated magnetic beads overnight at 4°C (Ge et al. 2020). Immunoprecipitated GFAP^+^ exosomes were washed with washing buffer and released from the beads using elution buffer.

### 4.18 Statistical Analyses

GraphPad Prism 8.0 software (GraphPad Software Inc., San Diego, CA, USA) and IBM SPSS Statistics version 26.0 (IBM Corp., Armonk, NY, USA) (Schroer et al. 2023) were used for carrying out data analysis. To compare the data from two independent groups, a two‐tailed unpaired Student's *t*‐test was utilized. For the comparison of data from three or more groups with a single factor, one‐way Analysis of Variance (ANOVA) followed by a Tukey post‐hoc test was applied. Two‐way ANOVA followed by a Tukey post‐hoc test was implemented for comparing two or more factors among multiple groups. Significance was considered when the *p*‐value was less than 0.05. Data analysis was conducted with GraphPad Prism 8.0. All data were displayed as the mean ± standard deviation (SD) (Liang et al. 2022). In the figure legends, the number of samples (*n*) and *p*‐values are all indicated.

## Author Contributions

**Yaru Liu:** writing – review and editing, writing – original draft, methodology, investigation, formal analysis, data curation, conceptualization. **Pan Liao:** writing – review and editing, writing – original draft, methodology, investigation, formal analysis, data curation, conceptualization. **Bo Yan:** methodology, data curation, conceptualization. **Dai Li:** methodology, data curation. **Shishuang Zhang:** methodology, data curation. **Wei Zhang:** methodology, data curation. **Zexi Jia:** methodology, data curation. **Zihan Zhang:** methodology, data curation. **Han Gao:** methodology, formal analysis. **Qiang Liu:** visualization, conceptualization. **Fanglian Chen:** writing – review and editing, visualization, validation, supervision, project administration, conceptualization. **Ping Lei:** writing – review and editing, visualization, validation, supervision, project administration, funding acquisition, conceptualization. **Zhenyu Yin:** writing – review and editing, writing – original draft, methodology, investigation, formal analysis, data curation, funding acquisition, conceptualization.

## Ethics Statement

All experimental protocols and procedures received approval from the Ethics Committee of Tianjin Medical University, aligning with National Institutes of Health guidelines.

## Conflicts of Interest

The authors declare no conflicts of interest.

## Supporting information


**Figure S1:** (A, B) Western blotting analysis of PSD95 in the A‐exo‐GFAP and A‐exo. Protein levels were normalized to GAPDH (*n* = 4 mice per group). (C–E) ELISA of TNF‐α, IL‐6 and IL‐1β, content in mouse brain tissue and blood (*n* = 5 mice per group). (F) Representative immunofluorescence staining of Iba1‐positive cells. Green indicates Iba1‐positive staining, and blue indicates positive DAPI nuclear staining. Scale bar = 50 μm. (G) Quantification of the number of Iba1 positive cell numbers (*n* = 4 mice per group). (Data were represented as the mean ± SD. *p < 0.05, **p < 0.01, and ****p < 0.0001.)

**Figure S2:** (A) Flow cytometry analysis of microglia (CD11b^+^CD45^int^) expressing P16, P53, BODIPY. (B) Quantification of P16, P53, BODIPY (*n* = 4 mice per group). (Data were represented as the mean ± SD. *p < 0.05, **p < 0.01, and ****p < 0.0001.)

## Data Availability

The data underlying the results of this study can be obtained from the corresponding author when a reasonable request is made.

## References

[acel70228-bib-0001] Alegre‐Zurano, L. , A. Garcia‐Baos , A. Castro‐Zavala , M. Medrano , I. Gallego‐Landin , and O. Valverde . 2023. “The FAAH Inhibitor URB597 Reduces Cocaine Intake During Conditioned Punishment and Mitigates Cocaine Seeking During Withdrawal.” Biomedicine & Pharmacotherapy 165: 115194. 10.1016/j.biopha.2023.115194. 37499453

[acel70228-bib-0002] Baeske, R. , T. Hall , R. R. Dall'Olmo , and M. F. Silva . 2024. “In People With Shoulder Pain, Mobilisation With Movement and Exercise Improves Function and Pain More Than Sham Mobilisation With Movement and Exercise: A Randomised Trial.” Journal of Physiotherapy 70, no. 4: 288–293. 10.1016/j.jphys.2024.08.009. 39327172

[acel70228-bib-0003] Bodart‐Santos, V. , L. S. Pinheiro , A. J. da Silva‐Junior , et al. 2023. “Alzheimer's Disease Brain‐Derived Extracellular Vesicles Reveal Altered Synapse‐Related Proteome and Induce Cognitive Impairment in Mice.” Alzheimer's & Dementia 19, no. 12: 5418–5436. 10.1002/alz.13134. 37204850

[acel70228-bib-0004] Chauquet, S. , E. F. Willis , L. Grice , et al. 2024. “Exercise Rejuvenates Microglia and Reverses T Cell Accumulation in the Aged Female Mouse Brain.” Aging Cell 23, no. 7: e14172. 10.1111/acel.14172. 38747044 PMC11258432

[acel70228-bib-0005] Cheng, F. , C. Wang , B. Yan , et al. 2024. “CSF1R Blockade Slows Progression of Cerebral Hemorrhage by Reducing Microglial Proliferation and Increasing Infiltration of CD8^+^CD122^+^ T Cells Into the Brain.” International Immunopharmacology 133: 112071. 10.1016/j.intimp.2024.112071. 38636374

[acel70228-bib-0006] Deczkowska, A. , H. Keren‐Shaul , A. Weiner , M. Colonna , M. Schwartz , and I. Amit . 2018. “Disease‐Associated Microglia: A Universal Immune Sensor of Neurodegeneration.” Cell 173, no. 5: 1073–1081. 10.1016/j.cell.2018.05.003. 29775591

[acel70228-bib-0007] Dumontet, C. , J. M. Reichert , P. D. Senter , J. M. Lambert , and A. Beck . 2023. “Antibody–Drug Conjugates Come of Age in Oncology.” Nature Reviews Drug Discovery 22, no. 8: 641–661. 10.1038/s41573-023-00709-2. 37308581

[acel70228-bib-0008] Elmore, M. R. P. , A. R. Najafi , M. A. Koike , et al. 2014. “Colony‐Stimulating Factor 1 Receptor Signaling Is Necessary for Microglia Viability, Unmasking a Microglia Progenitor Cell in the Adult Brain.” Neuron 82, no. 2: 380–397. 10.1016/j.neuron.2014.02.040. 24742461 PMC4161285

[acel70228-bib-0009] Garcia‐Martin, R. , G. Wang , B. B. Brandão , et al. 2021. “MicroRNA Sequence Codes for Small Extracellular Vesicle Release and Cellular Retention.” Nature 601, no. 7893: 446–451. 10.1038/s41586-021-04234-3. 34937935 PMC9035265

[acel70228-bib-0010] Ge, X. , M. Guo , T. Hu , et al. 2020. “Increased Microglial Exosomal miR‐124‐3p Alleviates Neurodegeneration and Improves Cognitive Outcome After rmTBI.” Molecular Therapy 28, no. 2: 503–522. 10.1016/j.ymthe.2019.11.017. 31843449 PMC7001001

[acel70228-bib-0011] Goetzl, E. J. , M. Mustapic , D. Kapogiannis , et al. 2016. “Cargo Proteins of Plasma Astrocyte‐Derived Exosomes in Alzheimer's Disease.” FASEB Journal 30, no. 11: 3853–3859. 10.1096/fj.201600756R. 27511944 PMC5067254

[acel70228-bib-0012] Goulielmaki, E. , A. Ioannidou , M. Tsekrekou , et al. 2020. “Tissue‐Infiltrating Macrophages Mediate an Exosome‐Based Metabolic Reprogramming Upon DNA Damage.” Nature Communications 11, no. 1: 42. 10.1038/s41467-019-13894-9. PMC6940362 31896748

[acel70228-bib-0013] Guo, M. , X. Ge , C. Wang , et al. 2023. “Intranasal Delivery of Gene‐Edited Microglial Exosomes Improves Neurological Outcomes After Intracerebral Hemorrhage by Regulating Neuroinflammation.” Brain Sciences 13, no. 4: 639. 10.3390/brainsci13040639. 37190604 PMC10137037

[acel70228-bib-0014] Hammond, T. R. , C. Dufort , L. Dissing‐Olesen , et al. 2019. “Single‐Cell RNA Sequencing of Microglia Throughout the Mouse Lifespan and in the Injured Brain Reveals Complex Cell‐State Changes.” Immunity 50, no. 1: 253–271. 10.1016/j.immuni.2018.11.004. 30471926 PMC6655561

[acel70228-bib-0015] Iwasa, K. , S. Yamamoto , K. Yamashina , et al. 2021. “A Peripheral Lipid Sensor GPR120 Remotely Contributes to Suppression of PGD2‐Microglia‐Provoked Neuroinflammation and Neurodegeneration in the Mouse Hippocampus.” Journal of Neuroinflammation 18, no. 1: 304. 10.1186/s12974-021-02361-2. 34961526 PMC8711188

[acel70228-bib-0016] Ji, X.‐Y. , Y.‐X. Guo , L.‐B. Wang , et al. 2024. “Microglia‐Derived Exosomes Modulate Myelin Regeneration via miR‐615‐5p/MYRF Axis.” Journal of Neuroinflammation 21, no. 1: 29. 10.1186/s12974-024-03019-5. 38246987 PMC10801965

[acel70228-bib-0017] Katz, P. S. , J. K. Sulzer , R. A. Impastato , S. X. Teng , E. K. Rogers , and P. E. Molina . 2015. “Endocannabinoid Degradation Inhibition Improves Neurobehavioral Function, Blood–Brain Barrier Integrity, and Neuroinflammation Following Mild Traumatic Brain Injury.” Journal of Neurotrauma 32, no. 5: 297–306. 10.1089/neu.2014.3508. 25166905 PMC4348366

[acel70228-bib-0018] Kihara, Y. , H. Mizuno , and J. Chun . 2015. “Lysophospholipid Receptors in Drug Discovery.” Experimental Cell Research 333, no. 2: 171–177. 10.1016/j.yexcr.2014.11.020. 25499971 PMC4408218

[acel70228-bib-0019] Kim, G. S. , E. Harmon , M. C. Gutierrez , et al. 2025. “Single‐Cell Analysis Identifies Ifi27l2a as a Gene Regulator of Microglial Inflammation in the Context of Aging and Stroke in Mice.” Nature Communications 16, no. 1: 1639. 10.1038/s41467-025-56847-1. PMC11828888 39953063

[acel70228-bib-0020] Langmann, T. , M. R. P. Elmore , R. J. Lee , B. L. West , and K. N. Green . 2015. “Characterizing Newly Repopulated Microglia in the Adult Mouse: Impacts on Animal Behavior, Cell Morphology, and Neuroinflammation.” PLoS One 10, no. 4: e0122912. 10.1371/journal.pone.0122912. 25849463 PMC4388515

[acel70228-bib-0021] Lee, K. S. , S. H. Yoon , I. Hwang , et al. 2024. “Hyperglycemia Enhances Brain Susceptibility to Lipopolysaccharide‐Induced Neuroinflammation via Astrocyte Reprogramming.” Journal of Neuroinflammation 21, no. 1: 137. 10.1186/s12974-024-03136-1. 38802820 PMC11131277

[acel70228-bib-0022] Lei, J. , X. Jiang , W. Li , et al. 2021. “Exosomes From Antler Stem Cells Alleviate Mesenchymal Stem Cell Senescence and Osteoarthritis.” Protein & Cell 13, no. 3: 220–226. 10.1007/s13238-021-00860-9. 34342820 PMC8901817

[acel70228-bib-0023] Li, L. , F. Li , X. Bai , et al. 2023. “Circulating Extracellular Vesicles From Patients With Traumatic Brain Injury Induce Cerebrovascular Endothelial Dysfunction.” Pharmacological Research 192: 106791. 10.1016/j.phrs.2023.106791. 37156450

[acel70228-bib-0024] Li, L. , R. Peng , C. Wang , et al. 2025. “β2 Integrin Regulates Neutrophil Trans Endothelial Migration Following Traumatic Brain Injury.” Cell Communication and Signaling 23, no. 1: 70. 10.1186/s12964-025-02071-9. 39923080 PMC11806581

[acel70228-bib-0025] Li, X. , Y. Li , Y. Jin , et al. 2023. “Transcriptional and Epigenetic Decoding of the Microglial Aging Process.” Nature Aging 3, no. 10: 1288–1311. 10.1038/s43587-023-00479-x. 37697166 PMC10570141

[acel70228-bib-0026] Liang, X. , W. Fa , N. Wang , et al. 2022. “Exosomal miR‐532‐5p Induced by Long‐Term Exercise Rescues Blood–Brain Barrier Function in 5XFAD Mice via Downregulation of EPHA4.” Aging Cell 22, no. 1: e13748. 10.1111/acel.13748. 36494892 PMC9835579

[acel70228-bib-0027] López‐Otín, C. , M. A. Blasco , L. Partridge , M. Serrano , and G. Kroemer . 2023. “Hallmarks of Aging: An Expanding Universe.” Cell 186, no. 2: 243–278. 10.1016/j.cell.2022.11.001. 36599349

[acel70228-bib-0028] Mohri, I. , M. Taniike , H. Taniguchi , et al. 2006. “Prostaglandin D2‐Mediated Microglia/Astrocyte Interaction Enhances Astrogliosis and Demyelination in Twitcher.” Journal of Neuroscience 26, no. 16: 4383–4393. 10.1523/jneurosci.4531-05.2006. 16624958 PMC6673986

[acel70228-bib-0029] Ruan, Z. , D. Pathak , S. Venkatesan Kalavai , et al. 2021. “Alzheimer's Disease Brain‐Derived Extracellular Vesicles Spread Tau Pathology in Interneurons.” Brain 144, no. 1: 288–309. 10.1093/brain/awaa376. 33246331 PMC7880668

[acel70228-bib-0030] Sang, D. , K. Lin , Y. Yang , et al. 2023. “Prolonged Sleep Deprivation Induces a Cytokine‐Storm‐Like Syndrome in Mammals.” Cell 186, no. 25: 5500–5516. 10.1016/j.cell.2023.10.025. 38016470

[acel70228-bib-0031] Santos‐Garcia, I. , C. Rodriguez‐Cueto , P. Villegas , et al. 2023. “Preclinical Investigation in FAAH Inhibition as a Neuroprotective Therapy for Frontotemporal Dementia Using TDP‐43 Transgenic Male Mice.” Journal of Neuroinflammation 20, no. 1: 108. 10.1186/s12974-023-02792-z. 37149645 PMC10163746

[acel70228-bib-0032] Schroer, A. B. , P. B. Ventura , J. Sucharov , et al. 2023. “Platelet Factors Attenuate Inflammation and Rescue Cognition in Ageing.” Nature 620, no. 7976: 1071–1079. 10.1038/s41586-023-06436-3. 37587343 PMC10468395

[acel70228-bib-0033] Spangenberg, E. E. , R. J. Lee , A. R. Najafi , et al. 2016. “Eliminating Microglia in Alzheimer's Mice Prevents Neuronal Loss Without Modulating Amyloid‐β Pathology.” Brain 139, no. Pt 4: 1265–1281. 10.1093/brain/aww016. 26921617 PMC5006229

[acel70228-bib-0034] Squillace, S. , and D. Salvemini . 2022. “Toll‐Like Receptor‐Mediated Neuroinflammation: Relevance for Cognitive Dysfunctions.” Trends in Pharmacological Sciences 43, no. 9: 726–739. 10.1016/j.tips.2022.05.004. 35753845 PMC9378500

[acel70228-bib-0035] Stark, A. K. , and J. S. Penn . 2024. “Prostanoid Signaling in Retinal Cells Elicits Inflammatory Responses Relevant to Early‐Stage Diabetic Retinopathy.” Journal of Neuroinflammation 21, no. 1: 329. 10.1186/s12974-024-03319-w. 39716241 PMC11667846

[acel70228-bib-0036] Subramanian, A. , P. Tamayo , V. K. Mootha , et al. 2005. “Gene Set Enrichment Analysis: A Knowledge‐Based Approach for Interpreting Genome‐Wide Expression Profiles.” Proceedings of the National Academy of Sciences of the United States of America 102, no. 43: 15545–15550. 16199517 10.1073/pnas.0506580102 PMC1239896

[acel70228-bib-0037] Thompson, A. G. , E. Gray , S. M. Heman‐Ackah , et al. 2016. “Extracellular Vesicles in Neurodegenerative Disease—Pathogenesis to Biomarkers.” Nature Reviews Neurology 12, no. 6: 346–357. 10.1038/nrneurol.2016.68. 27174238

[acel70228-bib-0038] Trotta, R. , S. Rivis , S. Zhao , et al. 2025. “Activated T Cells Break Tumor Immunosuppression by Macrophage Re‐Education.” Cancer Discovery 15: 1410–1436. 10.1158/2159-8290.Cd-24-0415. 40094380 PMC12223510

[acel70228-bib-0039] Verma, A. K. , J. Zheng , M. Mack , F. Ginhoux , S. Perlman , and D. E. Griffin . 2021. “Differential Effects of Prostaglandin D2 Signaling on Macrophages and Microglia in Murine Coronavirus Encephalomyelitis.” MBio 12, no. 5: 10‐1128. 10.1128/mBio.01969-21. PMC8546556 34488442

[acel70228-bib-0040] Waisman, A. , F. Ginhoux , M. Greter , and J. Bruttger . 2015. “Homeostasis of Microglia in the Adult Brain: Review of Novel Microglia Depletion Systems.” Trends in Immunology 36, no. 10: 625–636. 10.1016/j.it.2015.08.005. 26431940

[acel70228-bib-0041] Wallace, C. H. , G. Oliveros , P. A. Serrano , P. Rockwell , L. Xie , and M. Figueiredo‐Pereira . 2022. “Timapiprant, a Prostaglandin D2 Receptor Antagonist, Ameliorates Pathology in a Rat Alzheimer's Model.” Life Science Alliance 5, no. 12: e202201555. 10.26508/lsa.202201555. 36167438 PMC9515385

[acel70228-bib-0042] Wang, C. , V. Börger , A. Mohamud Yusuf , et al. 2022. “Postischemic Neuroprotection Associated With Anti‐Inflammatory Effects by Mesenchymal Stromal Cell‐Derived Small Extracellular Vesicles in Aged Mice.” Stroke 53, no. 1: e14–e18. 10.1161/strokeaha.121.035821. 34847707 PMC8700303

[acel70228-bib-0043] Wang, L. , H. Zhen , Y. Sun , et al. 2022. “Plasma Exo‐miRNAs Correlated With AD‐Related Factors of Chinese Individuals Involved in Aβ Accumulation and Cognition Decline.” Molecular Neurobiology 59, no. 11: 6790–6804. 10.1007/s12035-022-03012-0. 36040555 PMC9425792

[acel70228-bib-0044] Wong, L. R. , J. Zheng , K. Wilhelmsen , et al. 2022. “Eicosanoid Signalling Blockade Protects Middle‐Aged Mice From Severe COVID‐19.” Nature 605, no. 7908: 146–151. 10.1038/s41586-022-04630-3. 35314834 PMC9783543

[acel70228-bib-0045] Xiao‐Hang, Q. , C. Si‐Yue , and T. Hui‐Dong . 2024. “Multi‐Strain Probiotics Ameliorate Alzheimer's‐Like Cognitive Impairment and Pathological Changes Through the AKT/GSK‐3β Pathway in Senescence‐Accelerated Mouse Prone 8 Mice.” Brain, Behavior, and Immunity 119: 14–27. 10.1016/j.bbi.2024.03.031. 38548184

[acel70228-bib-0046] Yang, Q. , Y. Zhou , G. Jiang , et al. 2020. “Age‐Related CCL12 Aggravates Intracerebral Hemorrhage‐Induced Brain Injury via Recruitment of Macrophages and T Lymphocytes.” Aging and Disease 11, no. 5: 1103. 10.14336/ad.2019.1229. 33014526 PMC7505273

[acel70228-bib-0047] Yang, Z. J. , S. Y. Huang , K. Y. Zhong , et al. 2024. “Betaine Alleviates Cognitive Impairment Induced by Homocysteine Through Attenuating NLRP3‐Mediated Microglial Pyroptosis in an m(6)A‐YTHDF2‐Dependent Manner.” Redox Biology 69: 103026. 10.1016/j.redox.2024.103026. 38184996 PMC10808937

[acel70228-bib-0048] Yin, Z. , Z. Han , T. Hu , et al. 2020. “Neuron‐Derived Exosomes With High miR‐21‐5p Expression Promoted Polarization of M1 Microglia in Culture.” Brain, Behavior, and Immunity 83: 270–282. 10.1016/j.bbi.2019.11.004. 31707083

[acel70228-bib-0049] Yu, G. , L.‐G. Wang , Y. Han , and Q.‐Y. He . 2012. “clusterProfiler: An R Package for Comparing Biological Themes Among Gene Clusters.” OMICS: A Journal of Integrative Biology 16, no. 5: 284–287. 10.1089/omi.2011.0118. 22455463 PMC3339379

[acel70228-bib-0050] Zhang, H. , S. Lin , C. L. McElroy , et al. 2021. “Circulating Pro‐Inflammatory Exosomes Worsen Stroke Outcomes in Aging.” Circulation Research 129, no. 7: e121–e140. 10.1161/circresaha.121.318897. 34399581 PMC8448978

[acel70228-bib-0051] Zhang, T. , J. Yu , G. Wang , and R. Zhang . 2021. “Amyloid Precursor Protein Binds With TNFRSF21 to Induce Neural Inflammation in Alzheimer's Disease.” European Journal of Pharmaceutical Sciences 157: 105598. 10.1016/j.ejps.2020.105598. 33075465

[acel70228-bib-0052] Zhang, Y. , Y. Miao , J. Tan , F. Chen , P. Lei , and Q. Zhang . 2023. “Identification of Mitochondrial Related Signature Associated With Immune Microenvironment in Alzheimer's Disease.” Journal of Translational Medicine 21, no. 1: 458. 10.1186/s12967-023-04254-9. 37434203 PMC10334674

