# Supplementary figures and images for: DP1 Receptor Blockade Attenuates Microglial Senescence and Cognitive Decline Caused by PTGDS in Exosomes From Aged Brains
Source: Aging Cell. 2025 Sep 19;24(11):e70228. doi: 10.1111/acel.70228 (PMC12610410; doi:10.1111/acel.70228)

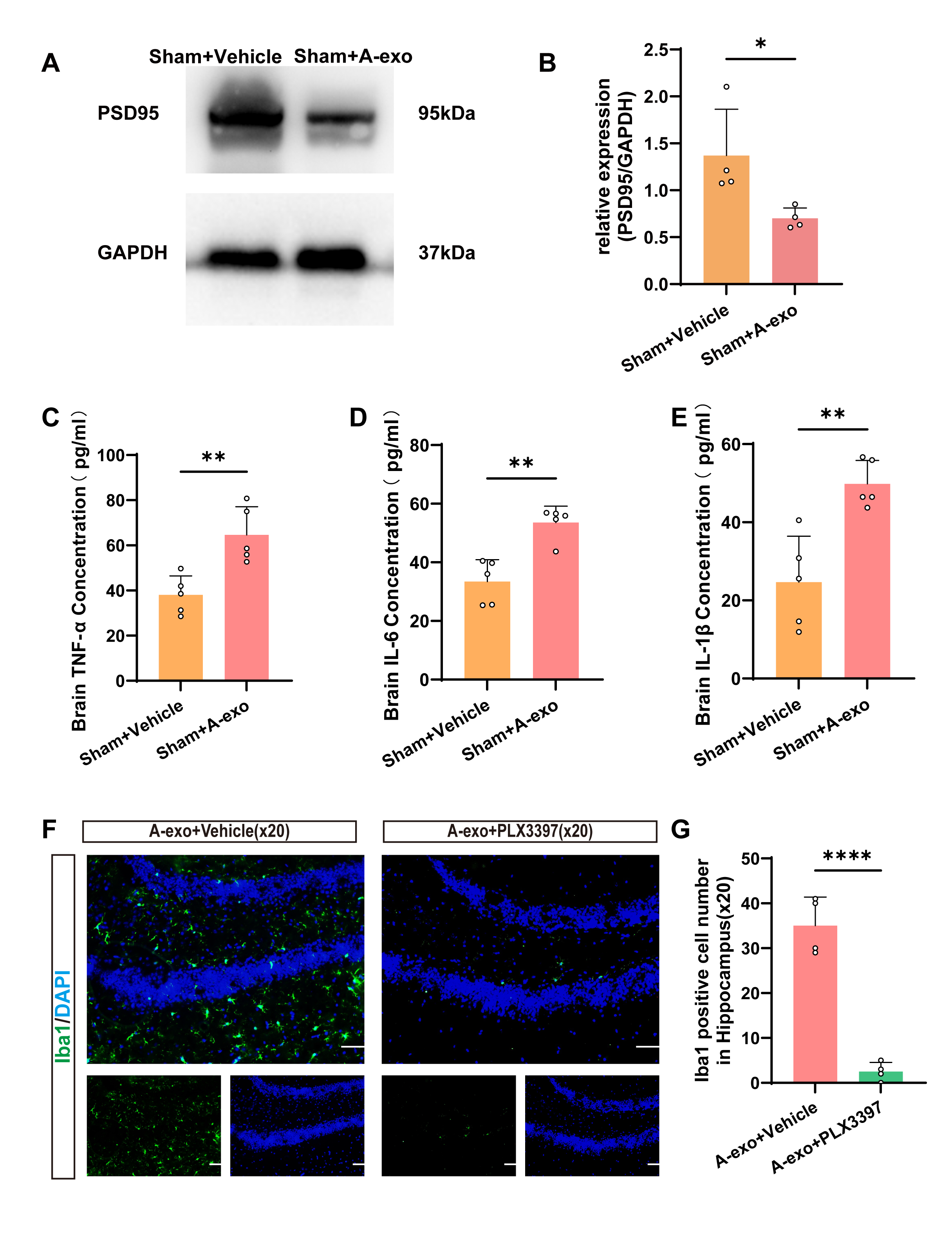

Supplement: Supplementary file 1 — Figure S1: (A, B) Western blotting analysis of PSD95 in the A‐exo‐GFAP and A‐exo. Protein levels were normalized to GAPDH (n = 4 mice per group). (C–E) ELISA of TNF‐α, IL‐6 and IL‐1β, content in mouse brain tissue and blood (n = 5 mice per group). (F) Representative immunofluorescence staining of Iba1‐positive cells. Green indicates Iba1‐positive staining, and blue indicates positive DAPI nuclear staining. Scale bar = 50 μm. (G) Quantification of the number of Iba1 positive cell numbers (n = 4 mice per group). (Data were represented as the mean ± SD. *p < 0.05, **p < 0.01, and ****p < 0.0001.) [file ACEL-24-e70228-s001.tif]

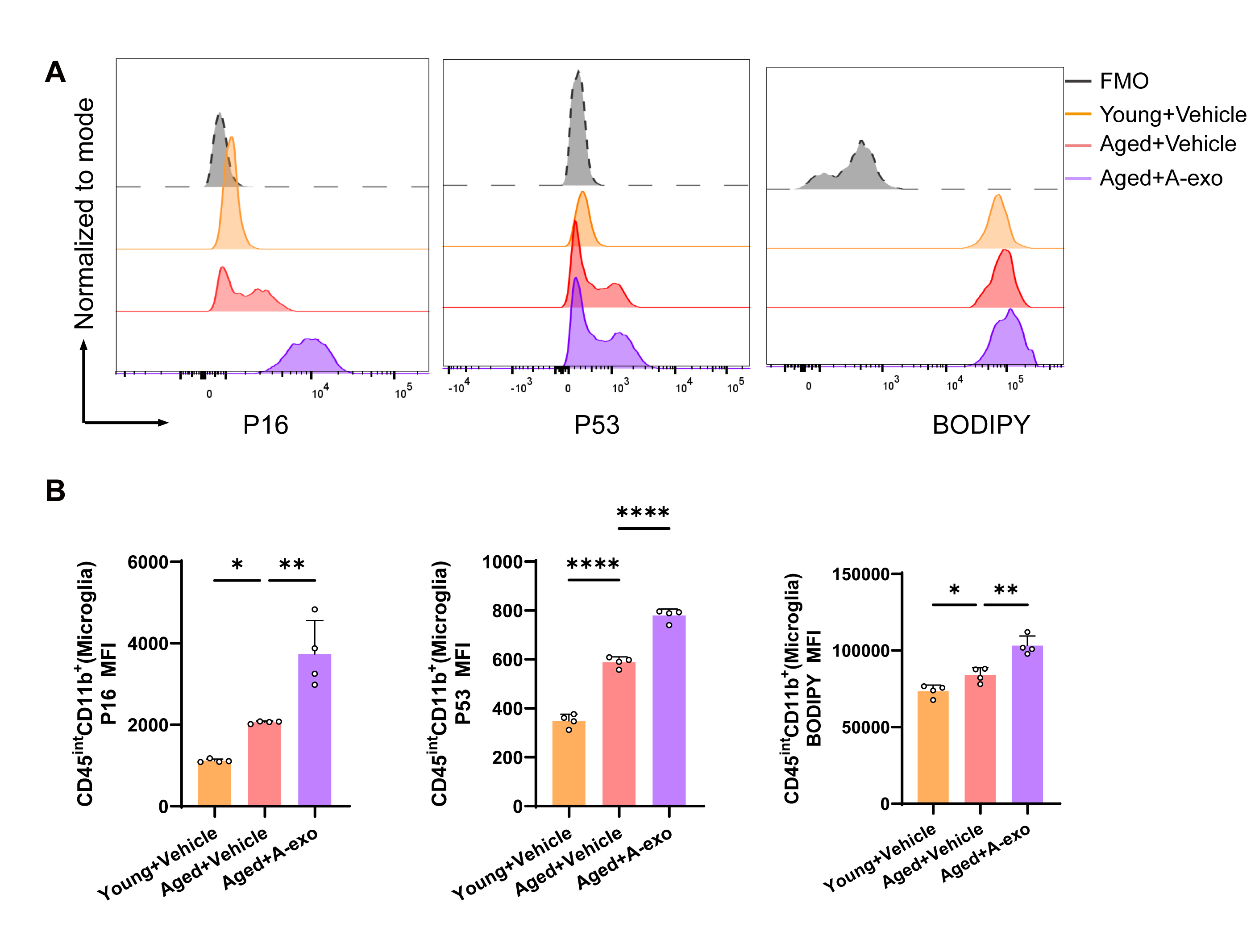

Supplement: Supplementary file 2 — Figure S2: (A) Flow cytometry analysis of microglia (CD11b+CD45int) expressing P16, P53, BODIPY. (B) Quantification of P16, P53, BODIPY (n = 4 mice per group). (Data were represented as the mean ± SD. *p < 0.05, **p < 0.01, and ****p < 0.0001.) [file ACEL-24-e70228-s002.tif]
